# Motor neuron translatome reveals deregulation of SYNGR4 and PLEKHB1 in mutant TDP-43 amyotrophic lateral sclerosis models

**DOI:** 10.1093/hmg/ddaa140

**Published:** 2020-07-07

**Authors:** Rita F Marques, Jan B Engler, Katrin Küchler, Ross A Jones, Thomas Lingner, Gabriela Salinas, Thomas H Gillingwater, Manuel A Friese, Kent E Duncan

**Affiliations:** Neuronal Translational Control Research Group, Center for Molecular Neurobiology Hamburg, University Medical Center Hamburg-Eppendorf, Hamburg 20251, Germany; Institute of Neuroimmunology and Multiple Sclerosis, Center for Molecular Neurobiology Hamburg, University Medical Center Hamburg-Eppendorf, Hamburg 20251, Germany; Neuronal Translational Control Research Group, Center for Molecular Neurobiology Hamburg, University Medical Center Hamburg-Eppendorf, Hamburg 20251, Germany; Edinburgh Medical School: Biomedical Sciences, University of Edinburgh, Edinburgh EH8 9XD, United Kingdom; NGS—Integrative Genomics Core Unit (NIG), Institute of Human Genetics, University Medical Center Göttingen, Göttingen 37077, Germany; NGS—Integrative Genomics Core Unit (NIG), Institute of Human Genetics, University Medical Center Göttingen, Göttingen 37077, Germany; Edinburgh Medical School: Biomedical Sciences, University of Edinburgh, Edinburgh EH8 9XD, United Kingdom; Institute of Neuroimmunology and Multiple Sclerosis, Center for Molecular Neurobiology Hamburg, University Medical Center Hamburg-Eppendorf, Hamburg 20251, Germany; Neuronal Translational Control Research Group, Center for Molecular Neurobiology Hamburg, University Medical Center Hamburg-Eppendorf, Hamburg 20251, Germany

## Abstract

Amyotrophic lateral sclerosis (ALS) is an incurable neurological disease with progressive loss of motor neuron (MN) function in the brain and spinal cord. Mutations in *TARDBP*, encoding the RNA-binding protein TDP-43, are one cause of ALS, and TDP-43 mislocalization in MNs is a key pathological feature of >95% of ALS cases. While numerous studies support altered RNA regulation by TDP-43 as a major cause of disease, specific changes within MNs that trigger disease onset remain unclear. Here, we combined translating ribosome affinity purification (TRAP) with RNA sequencing to identify molecular changes in spinal MNs of TDP-43–driven ALS at motor symptom onset. By comparing the MN translatome of *hTDP-43*^*A315T*^ mice to littermate controls and to mice expressing wild type *hTDP-43*, we identified hundreds of mRNAs that were selectively up- or downregulated in MNs. We validated the deregulated candidates *Tex26, Syngr4*, and *Plekhb1* mRNAs in an independent TRAP experiment. Moreover, by quantitative immunostaining of spinal cord MNs, we found corresponding protein level changes for SYNGR4 and PLEKHB1. We also observed these changes in spinal MNs of an independent ALS mouse model caused by a different patient mutant allele of TDP-43, suggesting that they are general features of TDP-43-driven ALS. Thus, we identified SYNGR4 and PLEKHB1 to be deregulated in MNs at motor symptom onset in TDP-43-driven ALS models. This spatial and temporal pattern suggests that these proteins could be functionally important for driving the transition to the symptomatic phase of the disease.

## Introduction

Amyotrophic lateral sclerosis (ALS, OMIM: #105400) is a progressive, adult-onset neurodegenerative disorder that mainly affects lower and upper motor neurons (MNs) in the spinal cord and brain, respectively (1–3). Disease usually begins with a focal weakness of a muscle, followed by a gradual spread to other muscles, leading to atrophy, paralysis, and death. The vast majority of patients die within 5 years after disease onset due to respiratory failure. Currently, only two therapies are approved for treating ALS: riluzole and edaravone, both of which only slow disease progression modestly (4–7). Thus, there is an urgent need for better therapeutics for this disease. ALS drug development would almost certainly be aided by a better understanding of the underlying molecular mechanisms that drive MN dysfunction. However, despite years of intensive investigation, it still remains unclear what molecular and cellular events in patient MNs lead to system breakdown after years of apparently normal function.

Major advances in ALS genetics have played a key role in helping to define molecular pathways that drive disease (8). As for other neurodegenerative diseases, ALS has both a sporadic form (sALS), in which no family history is defined, and familial forms (fALS), usually inherited as dominant traits, that account for ~10% of ALS cases (9–11). Modeling sporadic diseases in animals can be challenging, but the hope is that identifying genes that drive familial forms will enable robust modeling of all forms of disease. The first gene that was identified to cause ALS when mutated was superoxide dismutase 1 (*SOD1*), and for more than a decade, this was the only ALS gene that was known (12). Mouse models based on transgenic expression of mutant patient alleles of human *SOD1* develop adult-onset, progressive motor symptoms that are strikingly reminiscent of human ALS (13). Extensive work with these models has provided important insights into ALS pathobiology. For example, that complex interplay between many different cell types supports progression, but that disease nevertheless initiates within MNs themselves (14). This idea is supported by a more recent genome-wide study (15). Despite these advances, to date, many promising molecular targets identified using *SOD1* ALS models have failed to translate to helpful clinical therapies for human ALS. One possible explanation is that ALS caused by *SOD1* mutations might reflect a fundamentally different form of the disease in terms of its underlying molecular and cellular pathological mechanisms (9).

Several breakthrough discoveries in the last decade provided major new insight into ALS etiology. One was the discovery that TAR DNA-binding protein-43 (TDP-43) is a major component of protein aggregates frequently observed in postmortem brain and spinal cord of ALS patients, with the striking exception of ALS caused by mutations in *SOD1* (16,17). Soon thereafter, candidate gene studies identified mutations in the TAR DNA-binding protein (*TARDBP*) gene, which encodes the TDP-43 protein, in fALS and sALS cases (17–20, ALS10; OMIM: ^*^605078). Meanwhile, many other fALS genes have been identified, including repeat expansions in *C9ORF72* which are the most abundant genetic cause of ALS (21,22). Many fALS genes are also mutated in frontotemporal dementia (FTD), and these patients also have TDP-43 aggregates in affected cells, suggesting that ALS and FTD exist in a ‘disease continuum’ (23,24). Even though mutant TDP-43 causes a relatively small proportion of ALS, TDP-43 aggregates are present in the vast majority of ALS cases even when the *TARDBP* gene is not mutated. This makes TDP-43 a particularly interesting protein to study in the context of ALS and has led to the development of many cellular and animal models based mainly on the expression of patient mutant alleles (9,19,25).

Despite its name, TDP-43 is mainly characterized as an RNA-binding protein. It interacts with thousands of RNAs by preferentially binding to long UG repeats or UG-enriched RNA sequences (26–28) and regulates many post-transcriptional aspects of gene expression, including mRNA splicing, stability, transport, and translation (29). In addition to regulating mRNAs, TDP-43 has been implicated in microRNA processing and regulation, as well as long-non-coding RNA (ncRNA) expression (23,30). Although it is often assumed that TDP-43 aggregation compromises one of its normal functions in RNA metabolism, TDP-43 aggregates are not necessary for toxicity in numerous animal and cellular models of TDP-43-driven ALS (11,31). Nevertheless, TDP-43’s RNA-binding activity does appear to be required for toxicity (32). This supports a role for altered RNA regulation in TDP-43-driven ALS and raises the questions of which specific RNA regulatory functions are affected in disease and whether TDP-43 mutations cause disease by gain or loss of these functions. Analysis of post-mortem patient samples indicates that formation of cytoplasmic TDP-43 aggregates is often accompanied by depletion of TDP-43 from the nucleus (33–35). This observation has led to the proposal that loss of TDP-43’s nuclear function might be a key driver of disease. However, like aggregation, nuclear depletion of TDP-43 is also not required for toxicity (31). In addition, fALS patient mutations in TDP-43 show dominant inheritance and appear to preserve most cellular functions of the wild type (WT) protein, while simply overexpressing WT TDP-43 at a high enough level leads to motor symptoms in animals (29). Collectively, these observations appear to favor a gain-of-function mechanism but leave open which of TDP-43’s many functions and potential regulatory targets actually drive disease. As mutant TDP-43 overexpression specifically in MNs appears sufficient for disease in rodents (36), it seems likely that many disease-relevant alterations in RNA regulation will take place within MNs themselves. However, because TDP-43 is such a broad regulator of gene expression, any study that does not focus analysis specifically on these cells using a sensitive readout could miss the relevant targets.

Here, we reveal new proteins whose deregulation specifically within lower MNs correlates with the appearance of motor symptoms in mouse models of ALS driven by patient mutant variants of TDP-43. This was enabled by combining behavioral phenotyping across the disease time course with genome-wide gene expression analysis in MNs. Focusing on late asymptomatic and early symptomatic timepoints, we applied translating ribosome affinity purification (TRAP) to spinal MNs with consecutive RNA sequencing. Inclusion of animals that overexpress WT hTDP-43, but that do not develop symptoms, allowed us to filter out hundreds of changes in MN gene expression that do not correlate with disease. Two genes that passed this filter were *Syngr4* and *Plekhb1*, neither of which has previously been implicated in neurodegenerative diseases. Importantly, we show by quantitative analysis of spinal cord immunohistochemistry that the corresponding proteins synaptogyrin-4 (SYNGR4) and pleckstrin homology domain-containing family B member 1 (PLEKHB1) are respectively increased or decreased within MNs specifically during the transition from the asymptomatic to early symptomatic stage. Moreover, we observed similar changes in the levels of these proteins in a second TDP-43-dependant ALS mouse model driven by another TDP-43 patient mutation (Q331K). Collectively, our work identifies MN-specific regulatory targets of mutant TDP-43 in disease. The pattern of deregulation of these targets specifically at the transition to overt symptoms makes them promising candidates to drive the pathology within diseased MNs.

## Results

### Motor symptoms of disease appear between 8 and 12 weeks of age for female *Chat* bacTRAP; *hTDP-43^A315T^* double transgenic mice

Since the major goal of our study was to identify proteins whose levels would increase or decrease in MNs specifically coincident with disease symptom onset, we first sought to determine precisely the timing of the symptomatic transition in mice containing both the *Chat* bacTRAP (37) and *hTDP-43^A315T^* (38) transgenes. In addition, we wanted to explore whether the *Chat* bacTRAP transgene causes a motor phenotype on its own or modifies the disease phenotype of our transgenic hTDP-43 lines. Using three behavioral tests and body weight as readouts, we compared animals in age-matched cohorts of single and double transgenic mice to their WT littermates. We separately analyzed both males and females, since ALS shows sex differences in onset and progression in both humans and mouse models (39–41).

**Figure 1:**
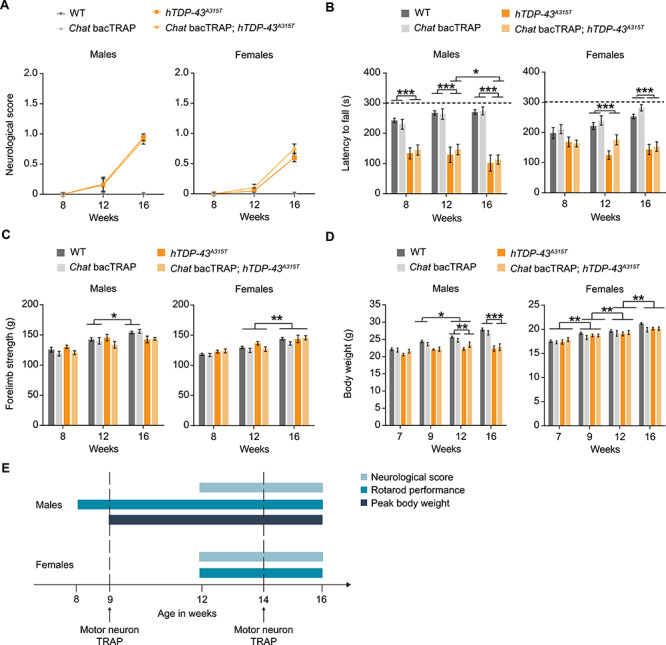
Behavioral assays reveal motor symptom onset in *Chat* bacTRAP*; hTDP-43^A315T^* ALS mice. (**A**) The ‘neurological score’ as assessed by tail-lifting and monitoring hindlimb extension. (**B**) Accelerating rotarod performance as measured by assessing latency to fall of the mice for a maximum of 300 s. (**C**) Forelimb grip strength was measured in multiples of gravitational force (g). (**D**) Body weight at indicated timepoints. (**E**) Schematic illustration of symptom onset timepoints. *n* = 8–12 per group; two-way RM ANOVA; Mean values ± SEM are shown; significance: ^^*^^P < 0.05; ^^*^^*^^P < 0.01 and ^^*^^*^^*^^P < 0.001.

Mice expressing the *hTDP-43^A315T^* transgene developed ALS-like motor phenotypes reflected in both the neurological score and accelerating rotarod tests (Fig. 1A and B). Importantly, the *Chat* bacTRAP transgene did not modify these phenotypes, and mice containing only this transgene were indistinguishable from WT controls (Fig. 1A–D). However, we noted differences between the sexes in timing of disease onset and severity. For neurological score, both sexes were asymptomatic at 8 weeks of age and began to show phenotypes at 12 weeks that worsen with time. In the accelerating rotarod test, which measures both muscle strength and coordination (42), male mice showed significant performance deficits already at 8 weeks of age, the earliest time point tested. In contrast, females were clearly asymptomatic on the rotarod at 8 weeks and only started to show similar phenotypes at 12 weeks of age (Fig. 1B), a time point coincident with the onset of symptoms in the neurological score test.

Unlike the neurological score and rotarod tests, forelimb grip strength did not show a statistically significant difference between genotypes, although males showed a trend toward a phenotype at 16 weeks (Fig. 1C). The absence of an effect on forelimb strength is consistent with the known focal onset of ALS in hind limbs of these mouse models and thus supports the notion that phenotypes in the other tests reflect an early stage of disease. Finally, although peak body weight has been suggested to be a marker for disease symptom onset in *hSOD1* models of ALS (43), it did not seem to be a consistent indicator for the mutant TDP-43 ALS model examined here. Male *hTDP-43^A315T^* transgenic mice reached their body-weight plateau at 9 weeks of age, while females gained weight equivalently to their control littermates throughout the experiment (Fig. 1D). Collectively, these data demonstrate that *hTDP-43^A315T^* male and female mice develop ALS-like motor phenotypes at different ages. Males show phenotypes in the rotarod at the earliest point tested and peak body weight shortly thereafter (8 and 9 weeks, respectively). In contrast, females show no phenotypes with any test at early time points and a clear transition from pre-symptomatic to early disease in the 9–12-week time window based on both neurological score and rotarod performance (Fig. 1E).

In addition to behavioral analysis, we independently confirmed the timing of disease symptom onset using a histological assessment of pathological changes occurring at the neuromuscular junction (NMJ). As shown in Supplementary Material, Figure S1, 14-week-old *hTDP-43^A315T^* mice showed a mild but statistically significant increase in the percentage of NMJs with abnormal morphology, reflecting the instigation/onset of pathological changes. No pathological NMJs were observed in WT control littermates. These results corroborate the early symptomatic phase of the disease at this age, and further suggest that NMJ synaptopathy could underlie the motor deficits observed in behavioral testing.

We also used behavioral testing to validate an important control line for our MN translatome assays that we refer to henceforth as *hTDP-43^WT^*. This line expresses WT human TDP-43 under the control of the same prion promoter as the *hTDP-43^A315T^* line, but reportedly does not develop any symptoms of ALS when hemizygous for the transgene (i.e. when only one chromosome is transgenic) (44). Using neurological score, rotarod performance, forelimb grip strength and body weight, we confirmed that *Chat* bacTRAP; *hTDP-43^WT^* double transgenic mice did not develop any symptoms of ALS and were indistinguishable from WT littermates in all three behavioral tests and by weight at every time point tested (Supplementary Material, Fig. S2). Thus, *Chat* bacTRAP; *hTDP-43^WT^* double transgenic mice provide an additional asymptomatic control line for MN translatome analyses. This control is important, since expression of WT hTDP-43 in mice at levels insufficient to cause disease is known to lead nevertheless to changes in gene expression (31). By including *Chat* bacTRAP; *hTDP-43^WT^* mice in our translatome assays, we can filter out genes whose altered expression in MNs is driven by hTDP-43 expression but does not correlate with disease.

### Major deregulation of the MN translatome is already apparent in early TDP-43-driven disease

**Figure 2:**
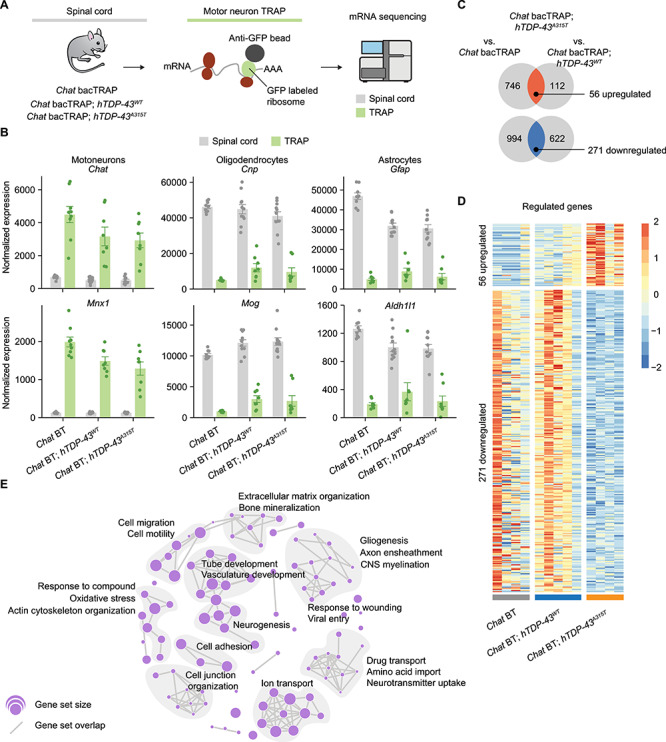
Screening of the motor neuronal translatome of *hTDP-43^A315T^* ALS mice after motor symptom onset. (**A**) Overview of the MN-TRAP experiment from three different genotypes: *Chat* bacTRAP, C*hat* bacTRAP*; hTDP-43^A315T^,* and *Chat* bacTRAP*; hTDP-43^WT^*. (**B**) Relative enrichment of MN genes (*Chat* and *Mnx1*) and de-enrichment of genes expressed in oligodendrocytes (*Cnp* and *Mog*) and astrocytes (*Gfap and Aldh1l1*) by MN-TRAP. Mean values ± SEM are shown. (**C**) Identification of differentially expressed (DE) genes in the MN translatome at 14 weeks. The Venn diagrams indicate the number of DE genes in the MN translatome of *Chat* bacTRAP*; hTDP-43^A315T^* mice in comparison to C*hat* bacTRAP and *Chat* bacTRAP*; hTDP-43^WT^* mice at 14 weeks, 56 upregulated and 271 downregulated genes were found to be exclusively deregulated in the *Chat* bacTRAP; *hTDP-43^A315T^* mice at 14 weeks. (**D**) Heatmap of genes found to be deregulated in *Chat* bacTRAP; *hTDP-43^A315T^* mice at 14 weeks. (**E**) Enrichment map of biological process GO terms. Node size represents gene set size, edges connect similar gene sets. Chat BT, *Chat* bacTRAP.

Having precisely defined the time window in which *Chat* bacTRAP; *hTDP-43^A315T^* mice become symptomatic, we next performed genome-wide MN-TRAP at corresponding time points (Fig. 2A). We focused on female animals, because they showed clear pre-symptomatic and symptomatic phases, whereas males did not (Fig. 1E). Prior to generating samples for mRNA sequencing, we first performed a series of assays to validate the performance of the *Chat* bacTRAP system under our specific experimental conditions. In particular, we wanted to verify efficient TRAP purification and MN expression in the double transgenic lines. Pilot TRAP purification assays with qRT-PCR as readout confirmed strong relative enrichment in MN-TRAP immunoprecipitated samples (IPs) of the motor neuronal marker *Chat* mRNA, and de-enrichment of non-motor neuronal mRNA *Gfap* in the presence and absence of the TDP-43 transgenes (Supplementary Material, Fig. S3A). Moreover, imaging of spinal cord sections from the transgenic animals revealed specific EGFP-L10a transgene expression that overlapped with the MN marker ChAT protein to a similar extent in all three transgenic genotypes (Supplementary Material, Fig. S3B–F). Consistent with previous reports, we observed EGFP-L10a signal in the MN cytoplasm and nucleolus (Supplementary Material, Fig. S3G). Together, these results demonstrate that the *Chat* bacTRAP transgene is similarly expressed in the different genotypes used here and can therefore be used to probe differences in the spinal MN translatome between them.

We next performed genome-wide RNA-Seq with MN-TRAP samples from spinal cord lysates of female *Chat* bacTRAP; *hTDP-43^A315T^* mice, at time points corresponding to pre- and early symptomatic phases of the disease (9- and 14-weeks-old, respectively; Fig. 2A). In parallel, we also performed MN-TRAP-Seq from cohorts of two age-matched, female control mouse lines: (1) *Chat* bacTRAP and (2) *Chat* bacTRAP; *hTDP-43^WT^* mice. For each sample, a fraction of the spinal cord lysate was retained as an ‘input control’ (IC) and analyzed in parallel to evaluate the sensitivity and specificity of MN-TRAP. We also tested ALS-like motor symptoms for all animals used in TRAP assays by applying the neurological score and body weight tests. As expected from our previous behavioral characterization, all 14-week old *Chat* bacTRAP; *hTDP-43^A315T^* female mice used for MN translatome analyses had difficulties in extension of the hind limbs, but showed no differences in body weight when compared to the *Chat* bacTRAP or *Chat* bacTRAP; *hTDP-43^WT^* control mice (Supplementary Material, Fig. S4A and B, respectively). Conversely, no significant differences in neurological score were detected at 9 weeks, confirming that *Chat* bacTRAP; *hTDP-43^A315T^* mice used for genome-wide TRAP assays were indeed pre-symptomatic at this time point.

In total, we analyzed 56 samples that successfully passed quality control (3–6 samples per group). In all samples, we observed robust enrichment of the motor neuronal marker mRNAs, *Chat* and *Mnx1* and de-enrichment of oligodendrocyte (*Cnp* and *Mog*) and glial markers (*Gfap* and *Aldh1l1*) across genotypes (Fig. 2B)*,* indicating that the TRAP methodology was efficient in capturing ribosome-associated mRNAs from spinal cord MNs. We first performed global analyses of our genome-wide data before proceeding with differential expression analysis. A cluster dendrogram revealed clear separation of samples based on whether they were spinal cord or MN-TRAP, as well as additional sub-clustering of most samples with the same genotype (Supplementary Material, Fig. S5A). Similarly, a principle component analysis (PCA) provides additional demonstration of a clear separation between MN and whole spinal cord (Supplementary Material, Fig. S5B). In comparison, differences based on genotype (Supplementary Material, Fig. S5C) or time point (Supplementary Material, Fig. S5D and E) appear subtler. Collectively, these global analyses of our MN translatome data demonstrate efficient purification of the MN translatome in all cases.

To identify specific de-regulated mRNAs, we focused on differences between genotypes at 14 weeks, which corresponds to the early symptomatic phase for *Chat* bacTRAP; *hTDP-43^A315T^* mice (Fig. 1). We first compared this line to *Chat* bacTRAP controls and identified 2067 deregulated genes (802 up and 1265 down). A second comparison to *Chat* bacTRAP; *hTDP-43^WT^* revealed 1061 genes that were deregulated (168 up and 893 down). The intersection of these two comparisons revealed a set of 327 genes that were either consistently upregulated or downregulated in the mutant line relative to both controls (56 up and 271 down; Fig. 2C; Supplementary Material, Table S3). Importantly, we detected very few changes between genotypes in the input spinal cord material, none of which correspond to the motor neuronal changes (Supplementary Material, Table S3). This underscores both the importance of focusing on specific cell types and the value of the TRAP method to identify changes that manifest specifically within the MN translatome.

To get a broad overview of biological processes that were affected in symptomatic *Chat* bacTRAP; *hTDP-43^A315T^* mice, we performed gene set enrichment analysis using the 327 identified deregulated genes as input. In total, we found 274 significantly regulated biological process gene ontology (GO) terms (Supplementary Material, Table S4), of which we used the top 100 to construct an enrichment map that clusters GO terms into biological themes (Fig 2E). Examples of those themes include ion transport, neurotransmitter uptake, CNS myelination, neurogenesis, oxidative stress, cell adhesion, and vasculature development, implying that diverse biological processes are perturbed in MNs already at an early stage in TDP-43-driven ALS.

To examine how changes to the MN translatome evolve over time, we used the same approach to analyze samples from 9-week-old mice, corresponding to the pre-symptomatic phase. At this timepoint, we found fewer differences, with only 109 mRNAs that were downregulated exclusively in the mutant (Supplementary Material, Fig. S6A, B, Supplementary Material, Table S5). Gene set enrichment analysis of these genes also identified many significantly regulated biological process GO terms at this timepoint (Supplementary Material, Table S6), enabling construction of an enrichment map with corresponding biological themes (Supplementary Material, Fig. S6C), some of which overlap with those found at 14 weeks (compare to Fig. 2E). Directly comparing the specifically deregulated genes in the MN translatome of pre-symptomatic *Chat* bacTRAP; *hTDP-43^A315T^* mice to symptomatic mice revealed 52 downregulated genes in common (Supplementary Material, Fig. S6D, Supplementary Material, Table S7). Thus, there are detectable changes exclusively in the MN translatome of TDP-43 mutant mice already at 9 weeks, about half of which appear to persist to 14 weeks. However, most of the changes that we observe in the MN translatome of symptomatic mice were not detected pre-symptomatically.

### Validation of *Tex26, Syngr4,* and *Plekhb1* mRNA level changes in the MN translatome of an independent cohort of *hTDP-43^A315T^* mice

**Figure 3:**
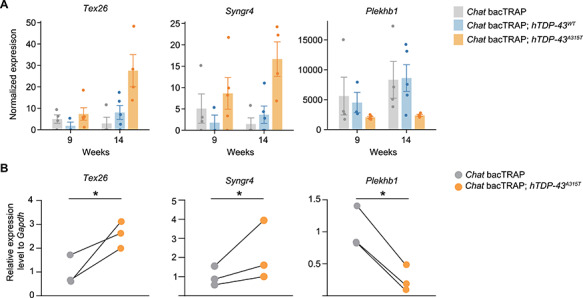
*Tex26*, *Syngr4* and *Plekhb1* mRNAs are deregulated in the *hTDP-43^A315T^* MN translatome at motor symptom onset. (**A**) RNA-Seq results for *Tex26*, *Syngr4,* and *Plekhb1* are shown at pre-symptomatic and early symptomatic stages (9 and 14 weeks, respectively). Gene expression levels are normalized to the sequencing library size. Mean values ± SEM are shown. (**B**) Corresponding qRT-PCR validation for *Tex26, Syngr4*, and *Plekhb1* mRNAs at 14 weeks. The qRT-PCR validation experiments are MN-TRAP assays from an independent cohort *Chat* bacTRAP*; hTDP-43^A315T^* and *Chat* bacTRAP control mice. Average relative expression values normalized to *Gapdh* mRNA levels are shown. *n* = 3 for qRT-PCR experiments; values for each genotype from each specific experiment are shown; corresponding genotype pairs from a specific replicate are linked by black lines; paired *t*-test; ^*^P < 0.05.

Out of the 327 genes that were exclusively de-regulated in the mutant line relative to both controls (Fig. 2C, Supplementary Material, Table S3), we selected four up- and two downregulated candidate disease drivers for further validation (Table 1). Our selection was driven by two criteria: (1) robust regulation in comparison to the control lines at 14 weeks, (2) upregulated genes should increase from pre-symptomatic to symptomatic timepoint (9–14 weeks). In follow-up analyses, we first sought to validate the MN-TRAP-Seq results in an independent cohort of *Chat* bacTRAP; *hTDP-43^A315T^* and singly transgenic *Chat* bacTRAP control mice. We performed MN-TRAP from spinal cords of three different 14-week-old animals for each genotype. Next, we checked our prioritized disease-associated candidates, obtaining reliable qRT-PCR data for four of the six candidates from Table 1. *Tex26* and *Syngr4* mRNA, which were upregulated in the MN-TRAP-Seq experiment (Fig. 3A and B), were also significantly upregulated in an independent MN-TRAP experiment (Fig. 3D and E). Moreover, *Plekhb1* mRNA was significantly downregulated, in line with the original MN-TRAP-Seq experiment (Fig. 3C and F). For two mRNAs, *Nhlh1* and *Mxd3* that were also upregulated in the MN-TRAP-Seq (Supplementary Material, Fig. S7), the qRT-PCR was not sensitive enough to enable validation, presumably due to very low expression levels (not shown). Only one tested mRNA, *Tia1*, showed no change, suggesting it might have been a false positive (Supplementary Material, Fig. S8). In summary, we validated three potential disease driver mRNAs in the MN translatome of *Chat* bacTRAP; *hTDP-43^A315T^* mice at disease symptom onset.

**Table 1:** Short list of upregulated and downregulated genes from the comparison of 14 weeks between the *Chat* bacTRAP; *TDP-43^A315T^* versus *Chat* bacTRAP; *hTDP-43^WT^* and *Chat* bacTRAP; *hTDP-43^A315T^* versus *Chat* bacTRAP, respectively

Type of regulation		*Chat* bacTRAP; *hTDP-43^A315T^* versus *Chat* bacTRAP		*Chat* bacTRAP; *hTDP-43^A315T^* versus *Chat* bacTRAP*; hTDP-43^WT^*	
	Gene name	Log_2_ fold change	padj	Log_2_ fold change	padj
Upregulated	*Tex26*	7.56	3.95E−05	2.7	3.10E−02
	*Syngr4*	5.19	4.80E−03	3.4	4.85E−02
	*Nhlh1*	8.83	6.30E−06	2.83	1.73E−02
	*Mxd3*	4.07	6.60E−03	3.41	1.54E−02
Downregulated	*Plekhb1*	3.17	4.2E−04	3.25	3.60E−04
	*Tia1*	2.84	6.90E–03	2.37	3.48E−02

### SYNGR4 and PLEKHB1 protein levels are altered in MNs of *hTDP43^A315T^* mice at disease onset

Having validated changes in levels in the MN translatome for several candidate disease driver mRNAs, we next asked whether changes in ribosome association with these mRNAs would have a functional impact on levels of the encoded proteins. Focusing on SYNGR4 and PLEKHB1, because antibodies were available, we performed IHC staining and confocal imaging of spinal cord sections from the lumbar area (~L3–5) of *hTDP-43^A315T^* and control mice at both 9 and 14 weeks of age. We quantified signal intensity within MN cell bodies in the ventral horn, after first confirming that this procedure gave significant signal over background relative to control samples lacking primary antibody for SYNGR4 (Supplementary Material, Fig. S9) or PLEKHB1 (Supplementary Material, Fig. S10). The disease phase of the animals used for these experiments was directly confirmed via neurological score. As expected, *hTDP-43^A315T^* mice used for IHC showed difficulties in hind limb extension at 14 weeks, reflecting early motor symptoms of ALS, but were pre-symptomatic at 9 weeks (Supplementary Material, Fig. S11).

**Figure 4:**
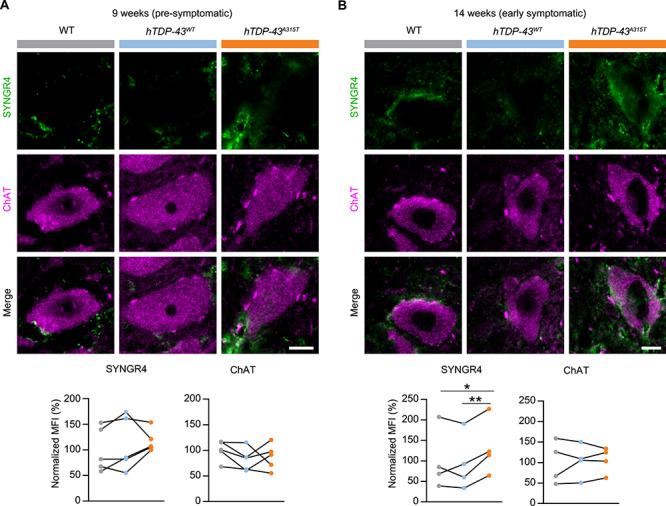
Increased SYNGR4 protein levels in spinal cord MNs of *hTDP-43^A315T^* ALS mice after disease onset. Immunofluorescent confocal images of SYNGR4 and ChAT co-staining of spinal cord MNs from L3–5 of *hTDP-43^A315T^* (A315T), WT and asymptomatic *hTDP-43^WT^* control (hTDP-43). Representative images are shown for 9-week-old (**A**) and 14-week-old (**B**) mice with quantification of mean fluorescent intensity (MFI). *n* = 4–5; values for each genotype from each specific experiment are shown; corresponding genotype pairs from a specific replicate are linked by black lines; paired *t*-test; scale bar: 10 μm.

**Figure 5:**
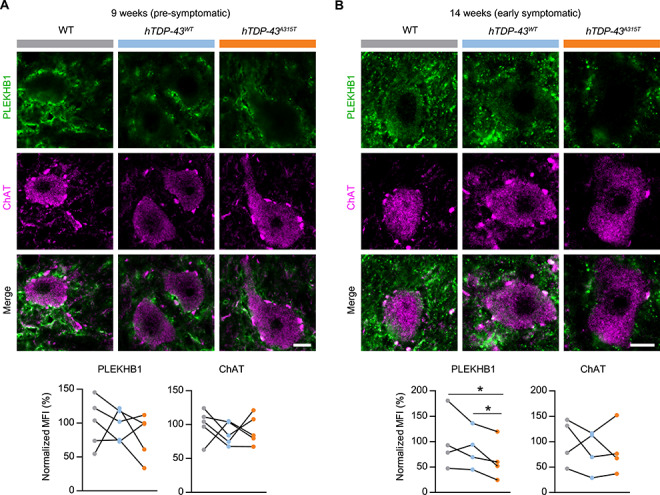
Decreased PLEKHB1 protein levels in spinal cord MNs of *hTDP-43^A315T^* ALS mice after disease onset. Immunofluorescent confocal images of PLEKHB1 and ChAT co-staining of spinal cord MNs from L3–5 of *hTDP-43^A315T^* (A315T), WT and the asymptomatic *hTDP-43^WT^* control (hTDP-43). Representative images are shown for 9-week-old (**A**) and 14-week-old (**B**) mice with quantification of mean fluorescent intensity (MFI). *n* = 4–5; values for each genotype from each specific experiment are shown; corresponding genotype pairs from a specific replicate are linked by black lines; paired *t*-test; ^*^P < 0.05; scale bar: 10 μm.

Comparison of fluorescent staining intensity in MN cell bodies during the pre-symptomatic phase revealed that levels of SYNGR4 protein were not significantly different between *hTDP-43^A315T^* mice and controls (9 weeks, Fig. 4A). However, in the early-symptomatic phase SYNGR4 protein levels were significantly increased in *hTDP-43^A315T^* MNs relative to both controls (14 weeks, Fig. 4B). Similar to SYNGR4, spinal cord MNs stained for PLEKHB1 during the pre-symptomatic phase revealed no significant differences between the *hTDP-43^A315T^* mice and controls (9 weeks; Fig. 5A). In contrast, during the early-symptomatic phase, PLEKHB1 protein levels in *hTDP-43^A315T^* MNs showed a decrease relative to both controls (Fig. 5B). Importantly, simultaneous staining for ChAT as an internal standard control revealed no significant alterations in its levels in either experiment (Figs 4 and 5), highlighting specific effects on SYNGR4 and PLEKHB1 protein levels, rather than general effects on MN protein levels or differential staining efficiency. Although SYNGR4 is related to two proteins found in synaptic vesicles (SVs) (45–47), we observed no obvious co-staining with a SV marker in spinal cord (Supplementary Material, Fig. S12). We conclude that SYNGR4 protein is upregulated and PLEKHB1 protein is downregulated within MNs of *hTDP-43^A315T^* mice in the transition from the pre-symptomatic phase to overt motor symptoms. The effects on SYNGR4 and PLEKHB1 protein levels in MNs are consistent with our MN translatome analyses (Fig. 3), which showed upregulation of *Syngr4* and downregulation of *Plekhb1* mRNAs specifically at the 14-week time point. Taken together, our results imply that changes in the levels of *Syngr4* and *Plekhb1* mRNAs in the MN translatome of *hTDP-43^A315T^* mice at disease onset lead to corresponding changes in the levels of these proteins within MNs.

### Deregulation of SYNGR4 and PLEKHB1 proteins in MNs of a different ALS-TDP model

In principle, effects on expression levels observed in MNs of *hTDP-43^A315T^* mice could be specific to the model used or a more general feature of ALS driven by altered TDP-43 function. To distinguish between these possibilities, we performed IHC for SYNGR4 and PLEKHB1 on spinal cords of an independent mouse ALS model caused by expressing TDP-43 with a different patient mutation: *hTDP-43^Q331K^* (31). This model was reported to develop the first ALS-like motor phenotypes when animals are 3 months old, but then does not progress further until after 6 months. Indeed, neurological scoring confirmed early disease for all *hTDP-43^Q331K^* mice at the time point used for IHC (21 weeks, Supplementary Material, Fig. S11).

**Figure 6:**
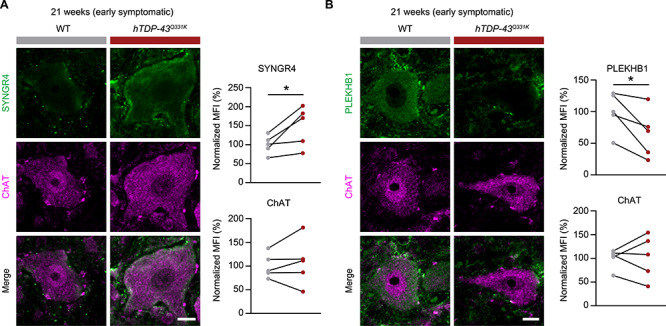
The *hTDP-43^Q331K^* ALS mouse model also shows increased SYNGR4 and decreased PLEKHB1 protein levels in spinal cord MNs early in disease. Representative immunofluorescent images of spinal cord MNs from L3–5 of *hTDP-43^Q331K^* (Q331K) or WT littermate controls are shown. All images are from 21-week-old mice. MNs were stained for SYNGR4 (**A**) or PLEKHB1 (**B**) and co-stained for ChAT as an internal control. Quantification of relative signal intensity is shown. MFI, mean fluorescence intensity. *n* = 5; scale bars: 10 μm; values for each genotype from each specific experiment are shown; corresponding genotype pairs from a specific replicate are linked by black lines; paired *t*-test; ^^*^^P < 0.05.

As for *hTDP-43^A315T^* mice, we quantified IHC signal intensity of spinal MNs in the lumbar area from 21-week-old *hTDP-43^Q331K^* mice and age-matched, WT control animals processed in parallel. We first confirmed a significant signal over background relative to control samples lacking primary antibody for both SYNGR4 and PLEKHB1 in this specific transgenic line and WT control littermates (Supplementary Material, Fig. S13). Having validated significant signal over background under our spinal MN staining conditions with these lines, we next quantified levels of the detected proteins in MN soma. SYNGR4 protein levels were also significantly upregulated in MNs of *hTDP-43^Q331K^* mice (Fig. 6A). Conversely, PLEKHB1 protein levels were significantly reduced in *hTDP-43^Q331K^* mice, as compared to WT controls (Fig. 6B). Simultaneous staining for ChAT, as an internal control, revealed no significant alterations in its levels, as expected (Fig. 6A and B). These results demonstrate that altered expression of SYNGR4 and PLEKHB1 proteins in MNs in early disease is not specific to a particular TDP-43 ALS model or patient mutation. Accordingly, they suggest that deregulation of these proteins in MNs may be a general, early event in mutant TDP-43-driven ALS.

## Discussion

To date there is no truly effective therapy for ALS. Consequently, almost all patients die within 5 years of diagnosis. One reason for this is that we do not yet fully understand the underlying molecular and cellular mechanisms that drive disease. Arguably, one of the most important issues to resolve is what molecular events mediate the transition to overt motor symptoms after a long pre-symptomatic period of apparently normal behavioral function. Here, we address this issue at the molecular level within MNs of mouse models of ALS caused by patient mutations in TDP-43. Using MN-TRAP, a cell-selective, genome-wide screening approach, we identified a set of mRNAs that show altered ribosome association in spinal MNs at the time when behavioral motor disease symptoms first appear. Subsequently, we validated changes in protein levels for two of these mRNAs, SYNGR4 and PLEKHB1, within MNs of two independent models of TDP-43-driven ALS. Because our results demonstrate that deregulation of these specific proteins correlates with the transition to overt motor symptoms, they reveal good candidates for molecular regulators of this fundamentally important transition in disease.

Several novel features of our experimental design enabled us to identify mRNAs and proteins that are deregulated within spinal cord MNs coincident with the transition to motor disease symptoms. First, we rigorously defined the timing of behavioral motor symptom onset in the specific lines used for MN-TRAP. This enabled us to identify optimal timepoints for the study and to identify tests that could be used reliably to verify disease phase for every animal within the TRAP experiment. These analyses also directly demonstrated that the *Chat* bacTRAP transgene had no modifying effect on the disease phenotypes and did not have a motor phenotype on its own. Second, we separately analyzed the two sexes. This proved to be crucial since males and females showed significantly different behavioral phenotypes. Most importantly, for the *hTDP-43^A315T^* line that we used, only females showed a clear pre-symptomatic phase and adult-onset transition to motor symptoms, as seen in human disease. Accordingly, we focused on females in our genome-wide MN-TRAP experiments. Third, we used RNA-Seq as a sensitive readout for our genome-wide TRAP assays. Finally, we included a key additional control: a congenic transgenic line that expresses WT hTDP-43 via the same promoter, but at a level insufficient to cause disease (44). We reasoned that this control could be crucial to identify mRNAs whose deregulation in MNs specifically correlates with disease. Indeed, we identified hundreds of TDP-43 responsive mRNAs that were deregulated in MNs of this asymptomatic transgenic hTDP-43 line and that were also deregulated at the same timepoint in the mutant showing disease symptoms. Thus, including this line in our analyses enabled us to filter out such mRNAs whose deregulation is clearly not sufficient to drive appearance of disease symptoms. The existence of many such mRNAs is consistent with a previous RNA-seq study with whole spinal cord that analyzed the impact of expression of hTDP-43 at levels insufficient to cause disease (31). However, our data extend this notion to ribosome-associated mRNAs in the translatome of MNs themselves.

With RNA-Seq as a sensitive readout, our analysis of the changes to MN translatome in the early symptomatic phase revealed several genes with differential expression between the *hTDP-43^A315T^* mice in comparison to both control genotypes. We believe this provides significant new insight beyond a previous microarray-based study which used a similar approach in a different TDP-43 mutant transgenic ALS model (48). That study only identified 19 endogenous coding mRNAs with significant changes between mutant and WT controls and did not include an asymptomatic hTDP-43 control. Global analyses of the deregulated genes that remain in our dataset after applying the asymptomatic hTDP-43 transgenic control filter revealed effects on several pathways that could contribute to motor neuronal function and health (Fig. 2D, Supplementary Material, Table S4).

By inspecting the regulation patterns for these specific mRNAs and applying additional filtering criteria, we obtained a short list of high-priority, temporally deregulated mRNAs (Table 1). What made these genes of particular interest for us is that their expression pattern seemed to robustly differ from the controls specifically at the symptomatic time point. In principle, altered expression of these genes within MNs might simply mark the transition to overt motor symptoms. However, it seems likely that changes in expression of specific genes will play an important functional role in driving the symptomatic transition. We propose that the temporal correlation between this transition and deregulation of these specific genes in MNs of our ALS models makes them promising candidates for proteins that have functional roles in driving the symptomatic transition.

How might deregulation of these specific genes drive the symptomatic transition? For TEX26, about which essentially nothing is known, it is impossible to say. However, NHLH1 and MXD3, encode basic helix-loop-helix family transcription factors (49,50), suggesting that deregulation of their transcriptional targets within MNs might conceivably contribute to the transition to overt symptoms. Testing this hypothesis will first require validation of changes in levels and activity of these proteins in MNs in disease. In the case of SYNGR4 and PLEKHB1, where we have verified protein level changes in MNs (Figs 4–6), it is also possible to form hypotheses for how these changes might contribute to altered MN physiology and thereby promote disease.

SYNGR4’s functions are unknown, but other synaptogyrin family members are well characterized. SYNGR1 and 3 are expressed mainly in neurons, where they are abundant components of SVs and are implicated in several aspects of the SV cycle, including neurotransmission itself (45–47). Intriguingly, SYNGR3 was recently reported to mediate pathological Tau protein recruitment to the pre-synapse in Alzheimer’s disease models (51). Nevertheless, there is no evidence that SYNGR4 is in SVs and we did not observe colocalization with an SV marker in MNs (Supplementary Material, Fig. S12). SYNGR4’s MN function might be more similar to those of ubiquitous SYNGR2/cellugyrin, which is a component of synaptic-like microvesicles (SLMVs) that is critical for their formation (45,52–54). SYNGR2 also reportedly promotes Bunyavirus infection via interaction with viral non-structural proteins (47) and other studies implicate SYNGR2 in intracellular vesicular protein transport (55). By analogy, SYNGR4 might also modulate the behavior of SLMVs in MNs and its upregulation at symptom onset might deleteriously impact motor neuronal function via this pathway.

PLEKHB1 (aka PKL1, evectin-1 or PHR1) is preferentially expressed in mammalian brain and sensory systems. In retina, it was shown to be an integral membrane protein found in the outer segment of both cones and rods (56). Membrane anchoring is mediated by the C-terminus, whereas the N-terminus contains a pleckstrin homology domain (PHD), which typically binds to phosphoinositides (PIPs) and/or G protein signaling subunits (57). PLEKHB1’s PHD domain appears not to bind to PIPs, as predicted based on its low lysine residue number. However, *in vitro* it bound to beta-gamma subunits of transducin, a key mediator of signal transduction in the visual system. Given that PLEKHB1 is broadly expressed outside the retina, the authors of this study suggested it might also bind to other G protein subunits in other CNS cell types. PLEKHB1’s PHD domain can also directly bind to myosin 1c and VIIa tails, which are suggested to play a role in anchoring the actin cytoskeleton to the plasma membrane of the vestibular and cochlear sensory cells (58,59). The PHD of this protein was also found to interact with CVB3 VP1, a major structural protein of coxsackievirus B3 (CVB3), which can cause CNS diseases including aseptic meningitis and encephalitis (60). Finally, based on its fractionation profile, it was also suggested that PLEKHB1 is a mediator of post-Golgi protein trafficking in cells that produce large amounts of membrane (61).

Taken together, previous studies of PLEKHB1 in other neuronal systems suggest a possible role in MNs in disease that would involve altered interactions with motor neuronal G-protein signaling complexes and/or myosin subunits at the plasma membrane and might in some way be related to viral infections affecting the CNS. Reduction of PLEKHB1 levels alone seems insufficient to trigger ALS-like motor symptoms: *Plekhb1* KO mice displayed no obvious phenotype when examined up to 1 year of age (62). However, this does not preclude a role for altered regulation of PLEKHB1 in controlling the timing of symptom onset during disease caused by mutant TDP-43. Future work should evaluate the impact of correcting the levels of both PLEKHB1 and SYNGR4 on the timing of symptom onset and progression. It will also be important to characterize their functions in MNs under both healthy and pathological conditions.

In summary, using a genome-wide screening approach, we have identified new genes and proteins which are deregulated in spinal cord MNs of TDP-43-driven ALS models coincident with the transition to overt disease symptoms. We observed upregulation of SYNGR4 and downregulation of PLEKHB1 proteins in MNs of two different models of TDP-43-driven ALS. Clearly, it would be interesting in the future to see whether deregulation of these proteins is a feature of all forms of ALS or only a subset. Indeed, an important, unresolved issue in ALS biology is whether diseases caused by different genetic lesions, nevertheless converge on a common set of molecular targets whose deregulation drives disease. In particular, it has been suggested that ALS caused by *SOD1* mutations might be different, as it does not feature the hallmark pathological aggregates of TDP-43 found in almost all other forms of ALS (63). A previous MN-TRAP analysis of a transgenic mouse model of ALS bearing a mutation in human *SOD1* also identified hundreds of genes deregulated in MNs of that model (15). While most of these did not show major overlap with those that we identified (~8%, 21 genes), such a comparison must be made with caution as the studies were performed in completely different labs at different times. Given the importance this issue has for both preclinical drug discovery and patient clinical trial design, we suggest that a future systematic, side-by-side comparison of carefully chosen ALS models is warranted. TDP-43 mutations also drive the related neurodegenerative disease, FTD, which exists in a disease spectrum with ALS (23,24). Moreover, TDP-43 proteinopathy has been suggested to be important in forms of amnestic dementia that mimic Alzheimer’s (so-called ‘LATE’) (64). It will be interesting to see whether SYNGR4, PLEKHB1 or perhaps other candidates from our list, are also deregulated in other affected neuronal populations during the age-dependent transition to overt symptoms in models of these other diseases. Systematic translatome profiling would clearly be informative here, and our work also highlights important elements that should be considered in the design of these studies.

## Materials and Methods

### Animal study approval

All animal care and experimental procedures were performed according to institutional guidelines and fully conformed to the requirements of the German Animal Welfare Act. Ethical approvals were obtained from the State Authority of Hamburg, Germany (approval nos. 83/15 and G14/003_Zucht Neuro).

### Mice

Mouse lines used in this study are indicated in Supplementary Material, Table S1. They were originally acquired from the Jackson Laboratory, Bar Harbor, Maine, USA. These lines were made congenic on the C57BL/6J background by backcrossing >10× at Jackson Labs and were maintained via backcrossing to C57BL/6J. All mice were kept under specific pathogen-free conditions except the ones undergoing behavior experiments in the ZMNH animal facility of the University Medical Center Hamburg-Eppendorf (UKE). We used mice (7–16 weeks old) from both sexes; mice were age-matched in all experiments.

### Behavior

For behavioral experiments, each tested group was initially composed of 8–12 animals of the same genotype and sex. Behavioral tests were conducted under the guidelines from ‘working with ALS mice: Guidelines for preclinical testing & colony management’, provided by the Jackson laboratory (65). Four tests were conducted on each group of animals: neurological score, peak body weight, grip strength and rotarod. The experiments were conducted in a dark room, when the animals were in their active phase of the day (awake stage).

Standard criteria were used to evaluate neurological score (65). Mice were held gently by the tail with their forelimbs grabbing the lid of the cage. Each mouse was tail-lifted for 5 s, and the experiment was conducted three times to assure the presence or absence of any symptoms. Both hindlimbs of each animal were assessed, and the corresponding average was calculated per time point tested. Animals were tested at 8, 12 and 16 weeks of age. For the peak body weight test, animals were weight at 7, 9, 12 and 16 weeks of age. Each animal was put on a calibrated empty cage on top of a balance. Regarding the forelimb grip strength, each animal was tested per time point three times. The final result per time point was calculated as the average of the three measurements. Animals were tested at 8, 12 and 16 weeks of age. Finally, for the rotarod, the mice were put on the rolling part of the device which was adjusted to a starting velocity of 7 rpm. The velocity increased with time to a final value of 34 rpm. The mice were left to run for a maximum of 300 s. Initially, the animals were given a habituation period of two trials followed by the real testing. Each mouse was tested three times per time point and an average of the three experiments per time point was done. Animals were tested at 8, 12 and 16 weeks of age.

Data analysis and statistics was done with Excel and GraphPad Prism 6 software, respectively. A two-way repeated measurement ANOVA (two-way RM ANOVA) test was used to calculate the significance among genotypes within the same time point and among genotypes at different time points. The Bonferroni’s *post hoc* test was used if a significant difference was found from the analysis of variance. Data is presented as mean ± SEM and ^*^, ^*^^*^ and ^*^^*^^*^ indicates a *P*-value of *P* < 0.05, *P* < 0.01 and *P* < 0.001, respectively.

### Muscle dissection and NMJ immunohistochemistry

Mice were anesthetized with a mixture of Ketamine (*Ketanest*^®^) (12 mg/mL) and Xylazine (*Rompun^®^*) (1.6 mg/mL) and after deep sleep induction were perfused with 4% paraformaldehyde (PFA). The hindlimbs were disconnected above the hip and the skin was carefully removed. Post-fixation of the hindlimbs was performed in 4% PFA solution for 1 h at room temperature (RT). The samples were washed with 1× PBS and all residual connective tissue was removed. The deep lumbrical muscles of the hindlimbs were dissected out and the NMJs were labelled using an established immunohistochemistry protocol in order to visualize pre-synaptic 2H3/SV2 proteins (medium weight neurofilament/SV protein) and post-synaptic acetylcholine receptors (AChRs) (66). Muscles were placed in tetramethylrhodamine-α bungarotoxin (TRITC-αBTX) (1:500, Biotium) for 30 min, followed by incubation with 4% Triton X in 1× PBS for 90 min, followed by a blocking solution of 2% Triton X and 4% bovine serum albumin in 1× PBS for 30 min. The primary antibodies, 2H3 and SV2 (both 1:50 mouse IgG, DSHB, AB 2314897 and AB 2315387, respectively) (made up in blocking solution) were incubated for 3 days at 4°C followed by 4 washes of 20 min with 1× PBS. The samples were then incubated with the secondary antibody, Alexa 488 (1:250 donkey anti-mouse IgG, Thermo Fisher Scientific, A-21202) (made up in 1× PBS) overnight at 4°C. Samples underwent final washing (4 washes of 20 min with 1× PBS) prior to whole mounting in Mowiol on glass slides. Slides were stored at −20°C prior to imaging. Quantification was performed on a standard fluorescence microscope. NMJ occupancy was judged as full, partial or vacant (on visual inspection) in addition to manual counting of axonal inputs per NMJ. 40 NMJs were assessed per muscle. Mean counts (% pathological endplates) for each group were compared by Mann-Whitney test (GraphPad, San Diego, CA).

### RNA isolation and purification via TRAP

The TRAP methodology was adapted from the original protocol from ‘Cell-Type-Specific mRNA Purification by Translating Ribosome Affinity Purification (TRAP)’ (37).

#### Affinity matrix preparation

Streptavidin MyOne T1 Dynabeads (Invitrogen) were incubated with 120 uL of Biotinylated Protein L (Fisher) for 35 min at RT on a rotator. The coated beads were washed 5× with 3% bovine serum albumin IgG and protease-free solution (Gibco) in PBS. The beads were re-suspended in 1 mL 0.15 M KCl buffer (20 mm HEPES, 150 mm KCl, 5 mm MgCl_2_, 1% NP-40, 0.5 mm DTT, 100 ug/mL cycloheximide, protease inhibitors, and RNase inhibitors) and 50 ug of 19C8 and 19F7 antibodies (Memorial Sloan Kettering Cancer Center, New York, USA) were added. The mixture was incubated at RT for 1 h on a rotator. The beads were washed with 0.15 M KCl buffer 3×, after which were re-suspended in 200 uL of 0.15 M KCl buffer. The IPs were stored on ice until the spinal cords were extracted and lysed.

#### Spinal cord extraction and lysis

Animals were anesthetized with a mixture of 80% of CO_2_ and 20% O_2_ prior being euthanized with 100% CO_2_. Spinal cords were removed and washed with dissection buffer (1× HBSS, 2.5 mm HEPES, 35 mm glucose, 4 mm sodium carbonate, 100 ug/mL cycloheximide (CHX)). The tissue was then transferred to a glass homogenizer in lysis buffer (20 mm HEPES, 150 mm KCl, 5 mm MgCl_2_, 0.5 mm DTT, 100 μg/mL CHX, protease inhibitors and RNase inhibitors). The homogenate was centrifuged at 4°C for 10 min at 2000 g to remove cellular debris. The supernatant was collected and kept on ice. NP-40 (Thermo Scientific) was added to a final concentration of 1% (v/v), followed by addition of dihexanoylphosphatidylcholine (DHPC, Avanti, final concentration 30 mm). The mixture was centrifuged at 4°C for 10 min at 20 000*g*. 10% of the supernatant was collected separately as an IC. The remaining 90% was incubated overnight at 4°C on a rotator with the 200 uL of the previously prepared beads. On the second day, the beads were washed 4× with 0.35 M KCl buffer (20 mm HEPES, 350 mm KCl, 5 mm MgCl_2_, 1% NP-40, 0.5 mm DTT, 100 μg/mL cycloheximide). After the final wash, the supernatant was discarded from the beads and 800 μL trizol (Life Technologies) was added to both IPs and ICs.

#### RNA clean-up and purification

The samples were incubated with 200 μL chloroform and shaken vigorously by hand for 15 s followed by 15 min centrifugation at 4°C, 12 000*g*. The upper phase was transferred to a fresh tube, and an equal amount of 100% EtOH was added. The RNA was purified using the PureLink RNA kit (Ambion). The purified RNA was treated with DNase I (Roche) to digest DNA, for 15 min at 37°C. The RNA was concentrated by an overnight sodium acetate precipitation (5 M sodium acetate (Sigma), 100% EtOH (Roth), GlycoBlue (Ambion) at −80°C. On the third day, the samples were removed from the −80°C freezer and directly centrifuged at 13 000*g* for 15 min at 4°C. The samples were washed twice with 70% EtOH with a spinning in between at 13 000*g* for 15 min at 4°C. The pellets were left to dry at RT for 15 min prior to being re-suspended in RNAse-free water (Ambion).

### qRT-PCR

500 ng of RNA from IC samples from TRAP experiments was used to make cDNA. In contrast, due to low RNA concentration from the IP samples from TRAP, a volume of 10 μL of RNA was used to make cDNA. cDNA synthesis was performed with random hexamer priming using the SuperScript^®^ II Reverse Transcriptase (Life Technologies) according to the manufacturer’s recommendation. Residual RNA was digested with RNase H (NEB) for 20 min at 37°C followed by enzyme inactivation by incubation for 10 min at 70°C. qPCR primers were either selected from the mouse primer depot website (www.mouseprimerdepot.nci.nih.gov) (Supplementary Material, Table S2). The cDNA was mixed with primers and FastStart Universal SYBR Green Master ROX solution (Roche). Each sample had three biological replicates. The real-time PCR was performed on an ABI 7900HT Fast Real-time PCR machine (Applied Biosystems). Analysis was performed using the ddCt method. Expression values were normalized to the control gene GAPDH or to the exogenous Spike-ins, as indicated. A minimum of three replicates was considered for statistical analysis. Excel was used to calculate the significance by applying a paired *t*-test. Data is presented as mean ± SEM and ^*^, ^*^^*^ and ^*^^*^^*^ indicates a *P*-value of *P* < 0.05, *P* < 0.01 and *P* < 0.001, respectively.

### RNA sequencing and translatome analysis

Samples were de-multiplexed with *bcl2fastq2* (Illumina, version 2.17) and sequencing quality control was performed using *FastQC* software (www.bioinformatics.babraham.ac.uk/projects/fastqc/). Sequencing reads were aligned to the Ensembl reference genome of *Mus musculus* (assembly version GRCm38, www.ensembl.org, (67) using the *STAR* software package (68, version 2.5.2b), with standard parameters. Quantification of gene expression was done with the *featureCounts* program (69, version 1.5.1), with standard parameters. Of all 62 sequenced samples, we excluded four samples with insufficient read counts and two samples that represented outliers in downstream analysis. The retained 56 samples were subjected to differential expression analysis with DESeq2 (70, version 1.14.1), calling genes with a minimal twofold change and false discovery rate (FDR)-adjusted *P* < 0.05 differentially expressed. Gene lists were annotated from BioMart using the package *biomaRt* (71, version 2.30.0). For subsequent analysis and visualization of count data, normalized expression values after variance stabilizing transformation (VST) in DESeq2 were used. Sample similarity was assessed by PCA using the top 500 most variable genes. Gene expression heatmaps were generated using the R package *pheatmap* (version 1.0.12). Scatter plots of individual genes were generated from normalized expression values without VST using the R package *ggplot2* (www.tidyverse.org, version 3.2.1).

### Candidate identification and gene set enrichment analysis

To identify potential disease-driving candidates, we compared symptomatic mutant animals versus two control lines at 14 weeks of age, e.g. (1) C*hat* bacTRAP*; hTDP-43^A315T^* versus C*hat* bacTRAP and (2) C*hat* bacTRAP*; hTDP-43^A315T^* versus C*hat* bacTRAP*; hTDP-43^WT^*. Candidates were required to be either consistently upregulated or downregulated in both comparisons (1 and 2). Candidate identifications was carried out for both the motor neuronal translatome (TRAP) and whole spinal cord samples (input control). With identified candidates from TRAP samples, a gene set enrichment analysis of biological process GO terms was performed using the online tool *ToppGene* (72, toppgene.cchmc.org). The top 100 significantly regulated GO terms were used to construct an enrichment map (73) that clusters GO terms into biological themes. For construction and plotting of the network, the R packages *tidygraph* (version 1.1.2.9999) and *ggraph* (version 2.0.0.9000) were used.

### Immunohistochemistry on spinal cord

The mice were anesthetized with a mixture of Ketamine (*Ketanest*^®^) (12 mg/mL) and *Xylazine* (*Rompun^®^*) (1.6 mg/mL) and after deep sleep induction they were perfused with 4% PFA (Roth) in PBS. Post-fixation of the spinal cord was done in 4% PFA solution for 2 h at RT, after which it was put in 30% sucrose solution in PBS until complete sank of the tissue. The tissue was then cut in three parts, corresponding to the cervical, thoracic and lumbar regions of the spinal cord and embedded in Tissue-Tek (Sakura) prior storage at −80°C.

Sections of the lumbar part of the spinal cord were further cut on a cryostat with a width of 12 μm. The tissue was blocked with a 10% normal donkey serum (Jackson Immuno Research) in 0.1% triton in PBS at RT for 45 min. The primary antibodies against EGFP (1:250, Abcam, ab13970), NeuN (1:400, Millipore, ABN91), PLEKHB1 (1:100, Biobyrt, orb326560), SYNGR4 (1:200, Aviva Systems Biology, OAPB01027), synaptophysin (1:500, Synaptic systems, 101 004) and ChAT (1:100, Millipore, AB144P) were diluted in 0.1% triton in PBS solution and left overnight at 4°C. On the next day, the slices were washed 3× with PBS and incubated with secondary antibodies (Alexa 647 and Alexa 488 (both 1:400, Jackson Immuno Research, 705-605-147 and 703-545-155, respectively), Alexa 555 (1:400, Abcam, ab150062)), in 1× PBS for 3 h at RT. After the incubation period, the slices were washed 3× with PBS. The slides were incubated with DAPI (1:1000, Thermo Fisher, #62248) in PBS for 10 min and embedded in roti mounting medium before sealing of the slide. The slices were stored at −20°C. Images were acquired from a Zeiss LSM 700 microscope and processed by ImageJ Fiji. A total of 5–6 images per experiment were taken, and a total of 4–5 age-matched animals per genotype were used.

Statistical analyses were performed using GraphPad Prism 6 software and a paired *t*-test. Data are presented as mean ± SEM, and ^*^, ^*^^*^ and ^*^^*^^*^ indicates a *P*-value of *P* < 0.05, *P* < 0.01 and *P* < 0.001, respectively.

## Author Contributions

R.F.M. planned and performed most experiments and analyzed the data; J.B.E. performed bioinformatic data analysis and interpreted results; K.K. helped to perform experiments; R.A.J. and T.H.G. planned, performed, analyzed and interpreted N.M.J. imaging experiments; T.L. and G.S.R. supervised RNA-seq library generation and performed bioinformatic data analysis; M.A.F. helped plan experiments and interpret results; K.E.D. conceived and supervised the project, planned experiments, helped to analyze data and interpreted results. K.E.D., R.F.M. and J.B.E. wrote the manuscript. All authors gave input on the manuscript and approved the final version.

## Supplementary Material

Marques_et_al_Supplementary_material-PDF_ddaa140

Supp_Table_3_Differential_gene_expression_14w_ddaa140

Supp_Table_4_Gene_set_enrichment_analysis_14w_ddaa140

Supp_Table_5_Differential_gene_expression_9w_ddaa140

Supp_Table_6_Gene_set_enrichment_analysis_9w_ddaa140

Supp_Table_7_Overlap_differential_gene_expression_between_9w_14w_ddaa140

CLEAN-Marques_et_al_Supplementary_material_ddaa140

HIGHLIGHTED-Marques_et_al_Supplementary_material_ddaa140
