# Supplementary material for: Motor neuron translatome reveals deregulation of SYNGR4 and PLEKHB1 in mutant TDP-43 amyotrophic lateral sclerosis models
Source: Hum Mol Genet. 2020 Jul 7;29(16):2647–61. doi: 10.1093/hmg/ddaa140 (PMC7530531; doi:10.1093/hmg/ddaa140)
Supplement: Marques_et_al_Supplementary_material-PDF_ddaa140 [file marques_et_al_supplementary_material-pdf_ddaa140.pdf]

SUPPLEMENTARY MATERIAL

SUPPLEMENTARY FIGURES

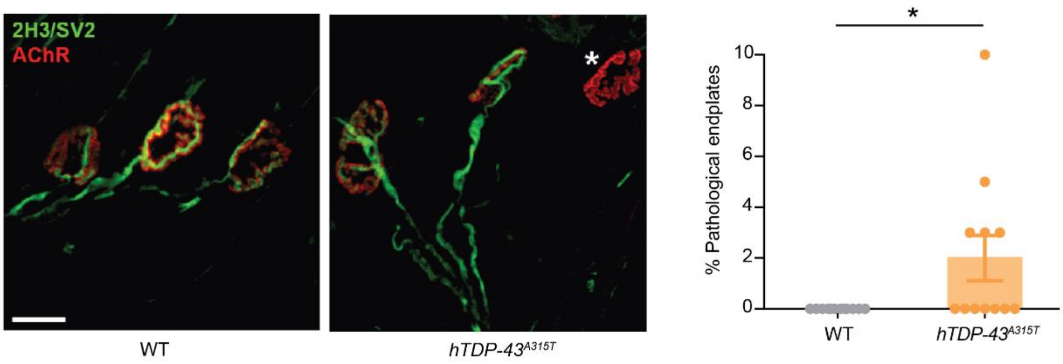

Supp. Fig. 1. The appearance of NMJ pathology correlates with symptom onset in *hTDP-43^{A315T}* ALS mice. Example representative confocal micrographs of neuromuscular junctions (NMJs) in hindlimb deep lumbrical muscles of 14-week-old mice (genotypes as indicated). NMJ pathology was observed in the *hTDP-43^{A315T}* mice, including markers of denervation (partially occupied and vacant endplates – white asterisk). Bar charts are mean ±SEM. Each data-point represents 1 muscle (average of 40 NMJs), n=6 animals per group (3 males and 3 females), 2 muscles per animal (left and right). Mann-Whitney test; *$p<0.05$, scale bar 20µm.

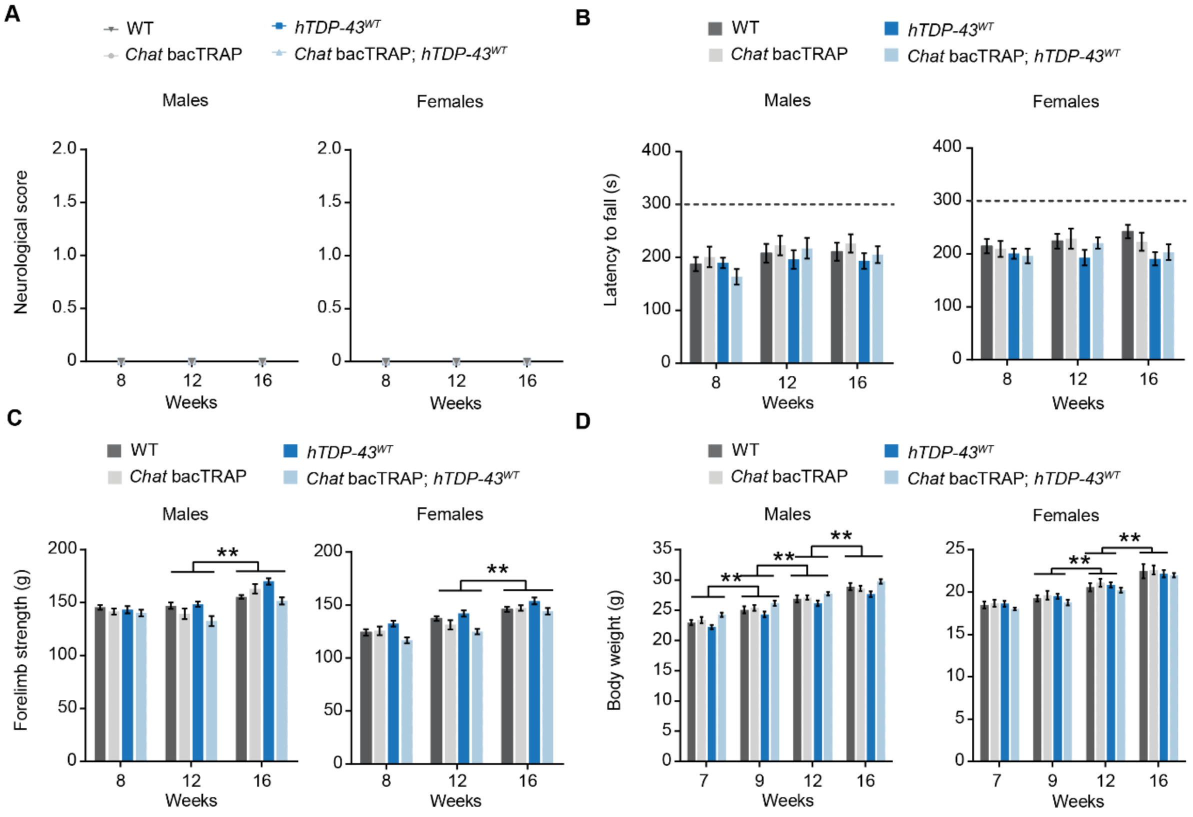

Supp. Fig. 2. *hTDP-43^{WT}* and *chat bacTRAP; hTDP-43^{WT}* mice do not develop ALS-like phenotypes. A cohort of *hTDP-43^{WT}* and *Chat* bacTRAP*; hTDP-43^{WT}* mice were tested in parallel with littermate controls as in Figure 1. **(A)** Neurological score, **(B)** accelerating rotarod performance, **(C)** forelimb grip strength and **(D)** average body weight. n= 8-12; 2-way RM ANOVA; Mean values +/- SEM are shown; significance: p<0.05* and p<0.01**.

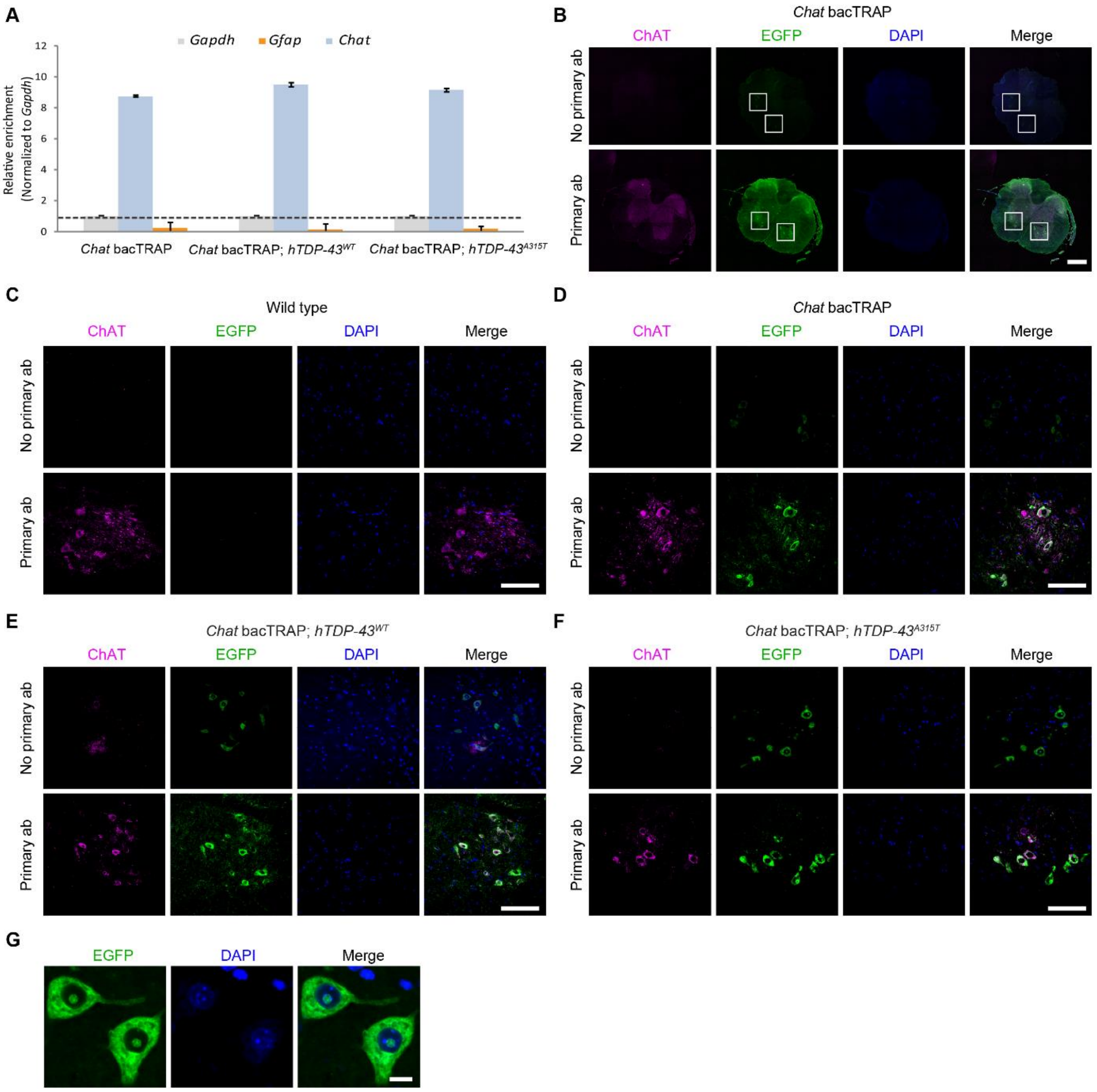

Supp. Fig. 3. *Chat* bacTRAP animals show specific EGFP-L10a protein expression in spinal cord motor neurons. (A) Relative enrichment of *Chat* mRNA and de-enrichment of *Gfap* mRNA from motor neurons from mouse spinal cord lysates. Mean values +/- SEM are shown. **(B)** Immunostaining of mouse spinal cord sections in the presence or absence of primary antibodies. Marked in white is the ventral horn, part of the spinal cord where motor neurons are located. Scale bar: 500µm. **(C, D, E and F)** Ventral horn staining of motor neurons for animals expressing EGFP-L10a protein under the *Chat* promoter and for animals non transgenic (WT). Experiments in the presence and absence of primary antibody were tested for each genotype. Scale bar: 50µm. **(G)** EGFP-L10a distribution in motor neurons. The protein can be found in both the nucleolus and the cell body. Scale bar: 50µm.

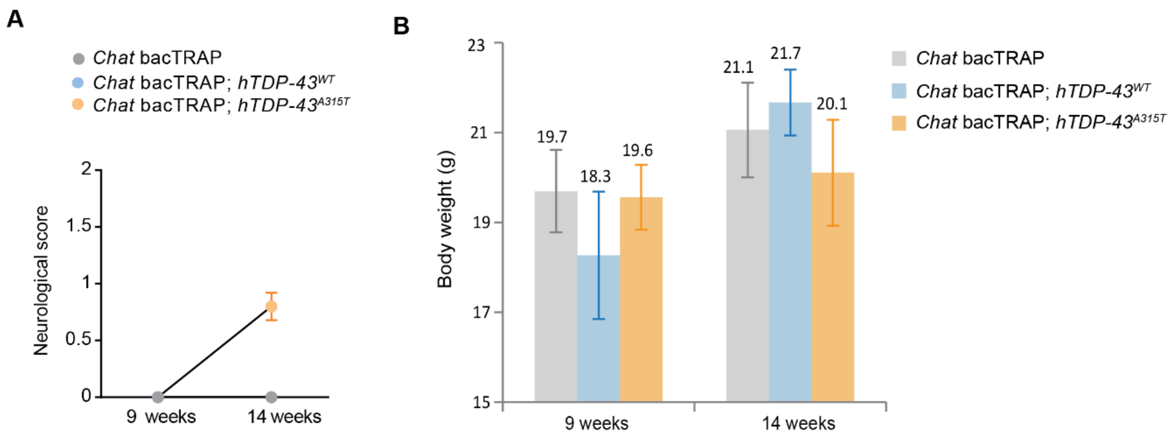

Supp. Fig. 4. Neurological phenotype of *Chat* bacTRAP; *hTDP-43^{A315T}* animals used for MN-TRAP-Seq experiments. (A) Average neurological score of mice used for genome-wide MN-TRAP-Seq experiments. The capacity for full hind leg extension was evaluated at 9 or 14 weeks, just prior to processing for TRAP. Mean values +/- SEM are shown. **(B)** Average body weight of mice used for genome-wide MN-TRAP-Seq experiments. n=5-6; mean values +/- SEM are shown.

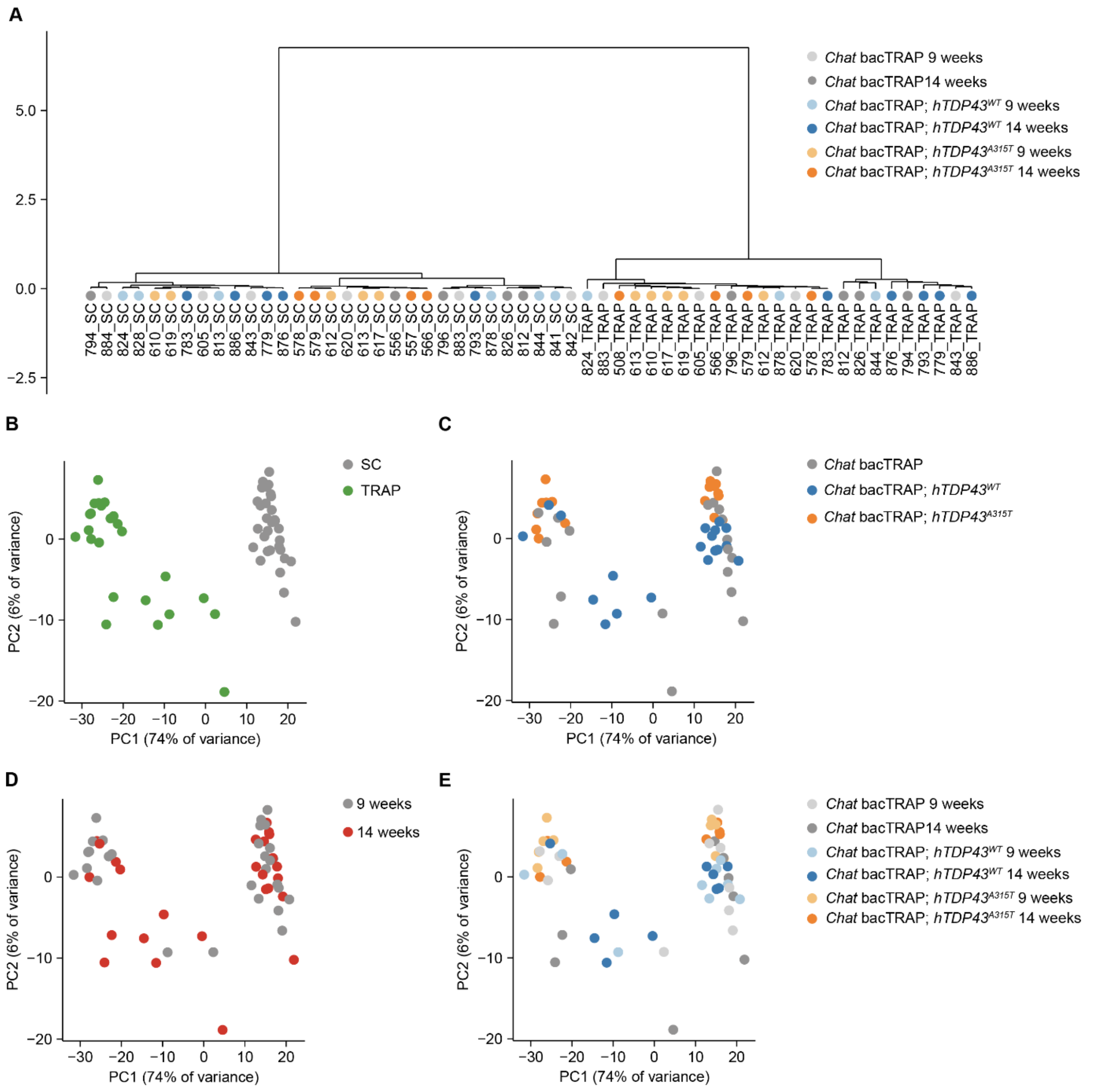

Supp. Fig. 5. Global analyses of the RNA-Seq data. (A) Dendrogram showing hierarchical clustering of analyzed samples. **(B)** A principal component analysis (PCA) plot colored by **(B)** sample type, **(C)** genotype, **(D)** timepoint and **(E)** a combination of genotype and timepoint.

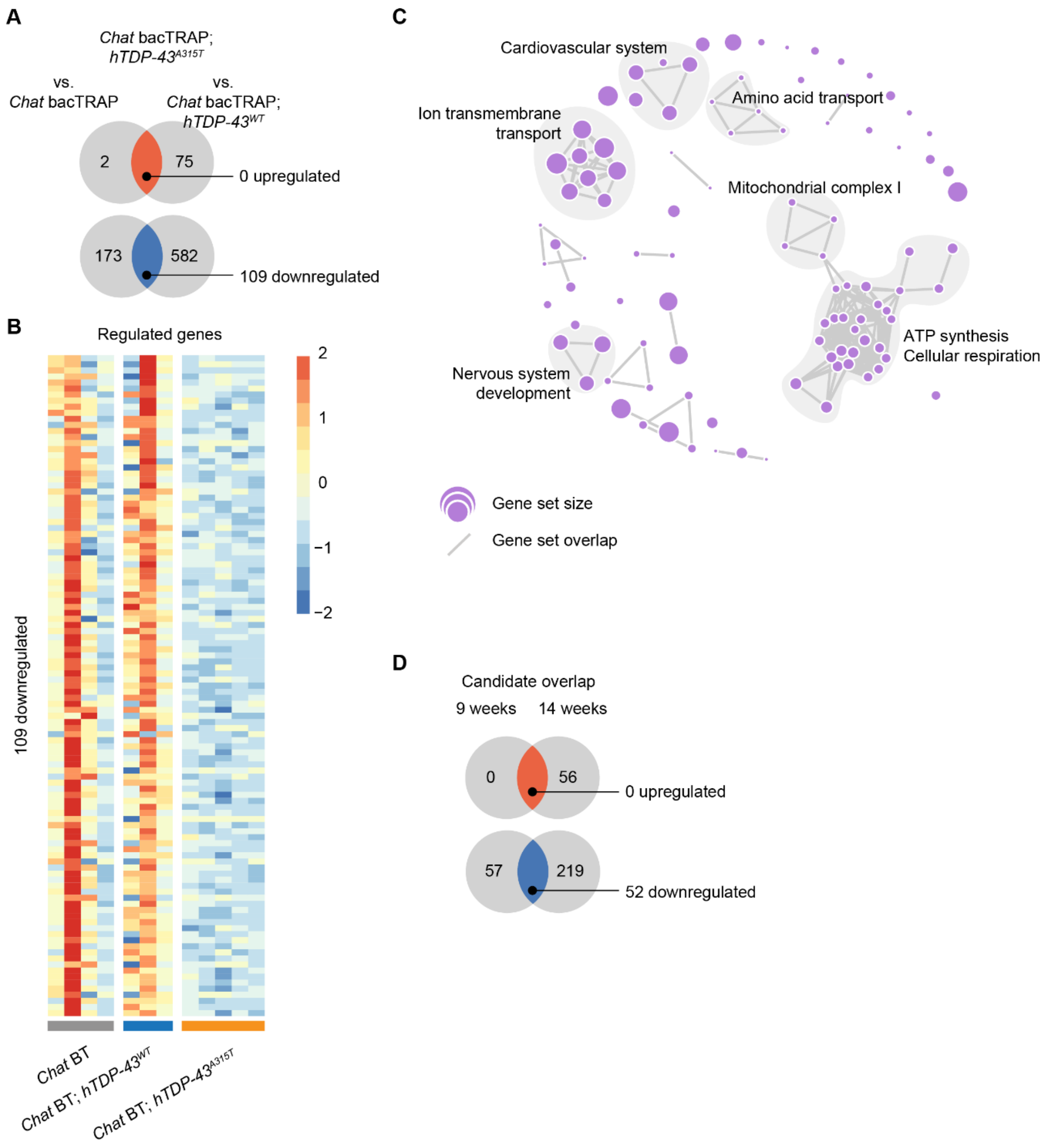

Supp. Fig. 6. Screening of the motor neuronal translatome of *hTDP-43*^{A315T} ALS mice before motor symptom onset. (A) Identification of differentially expressed (DE) genes in the MN translatome at 9 weeks. Venn diagrams indicate the number of DE genes in the MN translatome of *Chat* bacTRAP*; hTDP-43*^{A315T} mice in comparison to C*hat* bacTRAP and *Chat* bacTRAP*; hTDP-43*^{WT} mice at 9 weeks. 0 upregulated and 109 downregulated genes were found to be exclusively deregulated in the *Chat* bacTRAP; *hTDP-43*^{A315T} mice at 9 weeks. **(B)** Heatmap of genes found to be deregulated in *Chat* bacTRAP; *hTDP-43*^{A315T} mice at 9 weeks. **(C)** Enrichment map of biological process gene ontology (GO) terms. Node size represents gene set size, edges connect similar gene sets. Chat BT: *Chat*

bacTRAP. **(D)** Identification of DE overlapping genes between 9 and 14 weeks. 52 downregulated genes were found to overlap between both timepoints.

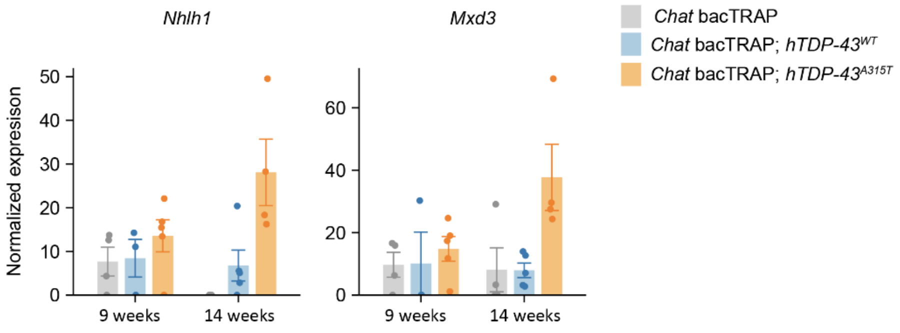

Supp. Fig. 7. *Nhlh1* and *Mxd3* mRNAs are deregulated in the *hTDP-43^{A315T}* MN translatome at motor symptom onset. MN-TRAP sequencing results for the genes *Nhlh1* and *Mxd3* in the indicated genotypes are shown at pre-symptomatic and early symptomatic stages (9 and 14 weeks, respectively). Gene expression levels are normalized to total gene counts. Mean values +/- SEM are shown.

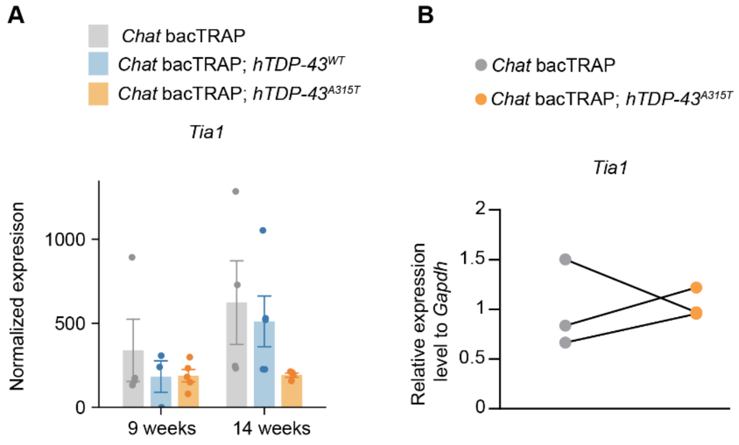

Supp. Fig. 8. No change in Tia1 mRNA levels in MN-TRAP validation experiment. (A) MN-TRAP sequencing results for the gene *Tia1* at 14 weeks are shown. Mean values +/- SEM are shown. **(B)** qRT-PCR data for *Tia1* MN-TRAP at 14 weeks with an independent cohort are shown. n=3; paired t-test; p>0.05.

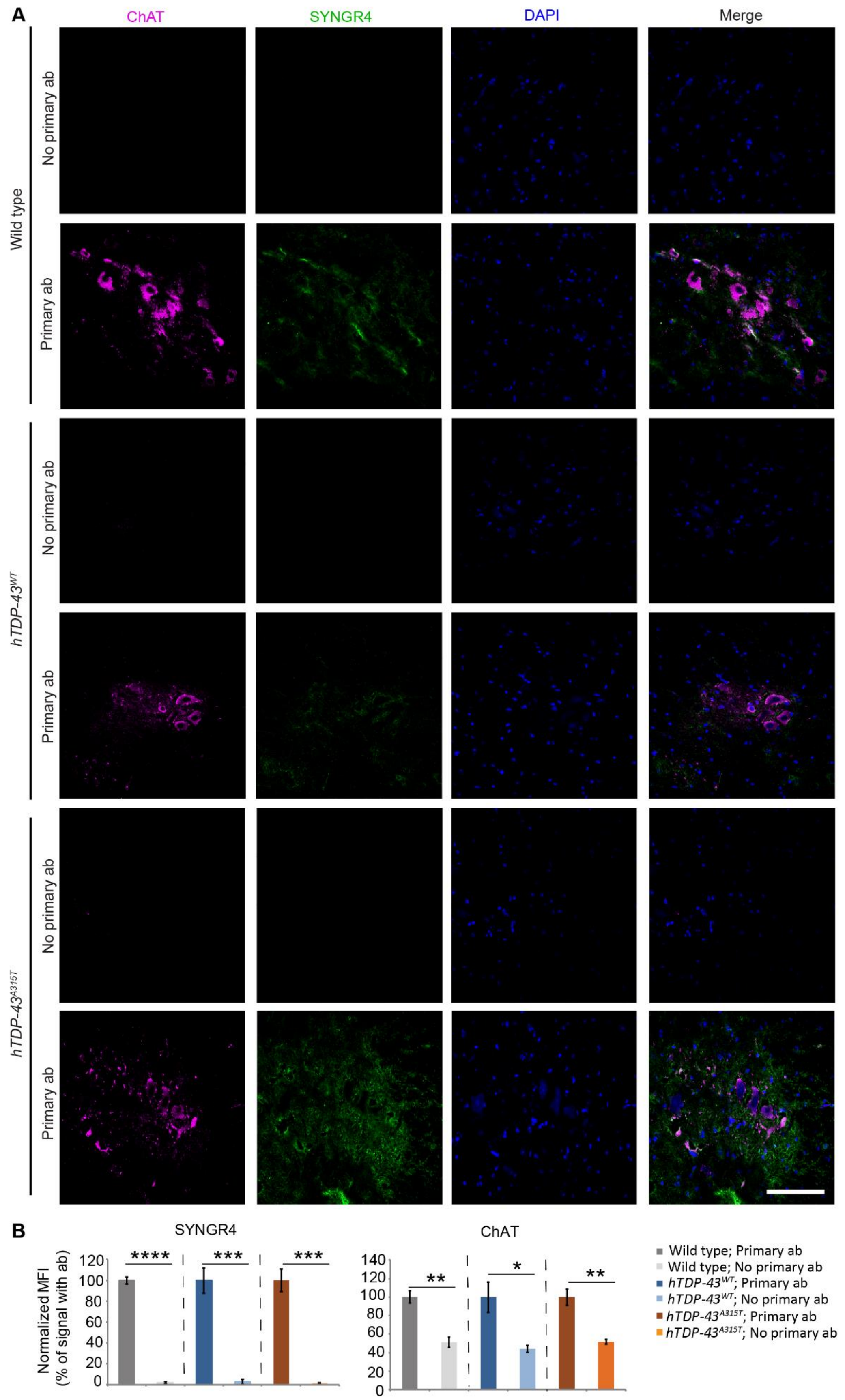

A
ChAT
SYNGR4
DAPI
Merge
Wild type
No primary ab
Primary ab
hTDP-43^WT
No primary ab
Primary ab
hTDP-43^A315T
No primary ab
Primary ab
B
SYNGR4
Normalized MFI (% of signal with ab)
0
20
40
60
80
100
120
****
***
***
ChAT
0
20
40
60
80
100
120
140
**
*
**
Wild type; Primary ab
Wild type; No primary ab
hTDP-43^WT; Primary ab
hTDP-43^WT; No primary ab
hTDP-43^A315T; Primary ab
hTDP-43^A315T; No primary ab

Supp. Fig. 9. SYNGR4 protein staining is primary antibody dependent. (A) Spinal cord staining of wild type, *hTDP-43*^{WT} and *hTDP-43*^{A315T}. Animals were tested for signal to noise (background) levels in the absence of primary antibodies. Images are representative of n=3 experiments. **(B)** Quantification of signal to noise for SYNGR4 and ChAT antibodies. For each genotype, values for no primary control images (noise) are indicated as a percentage of signal in the presence of primary antibody (signal). All antibodies show significantly higher mean fluorescence intensity (MFI) when compared to the respective no primary antibody control. n=3 independent biological replicates; ab: antibody; two-tailed, two sample t-test; mean values +/- SEM are shown; *p<0.05, **p<0.01, ***p<0.001 and ****p<0.0001; scale bar: 10μm.

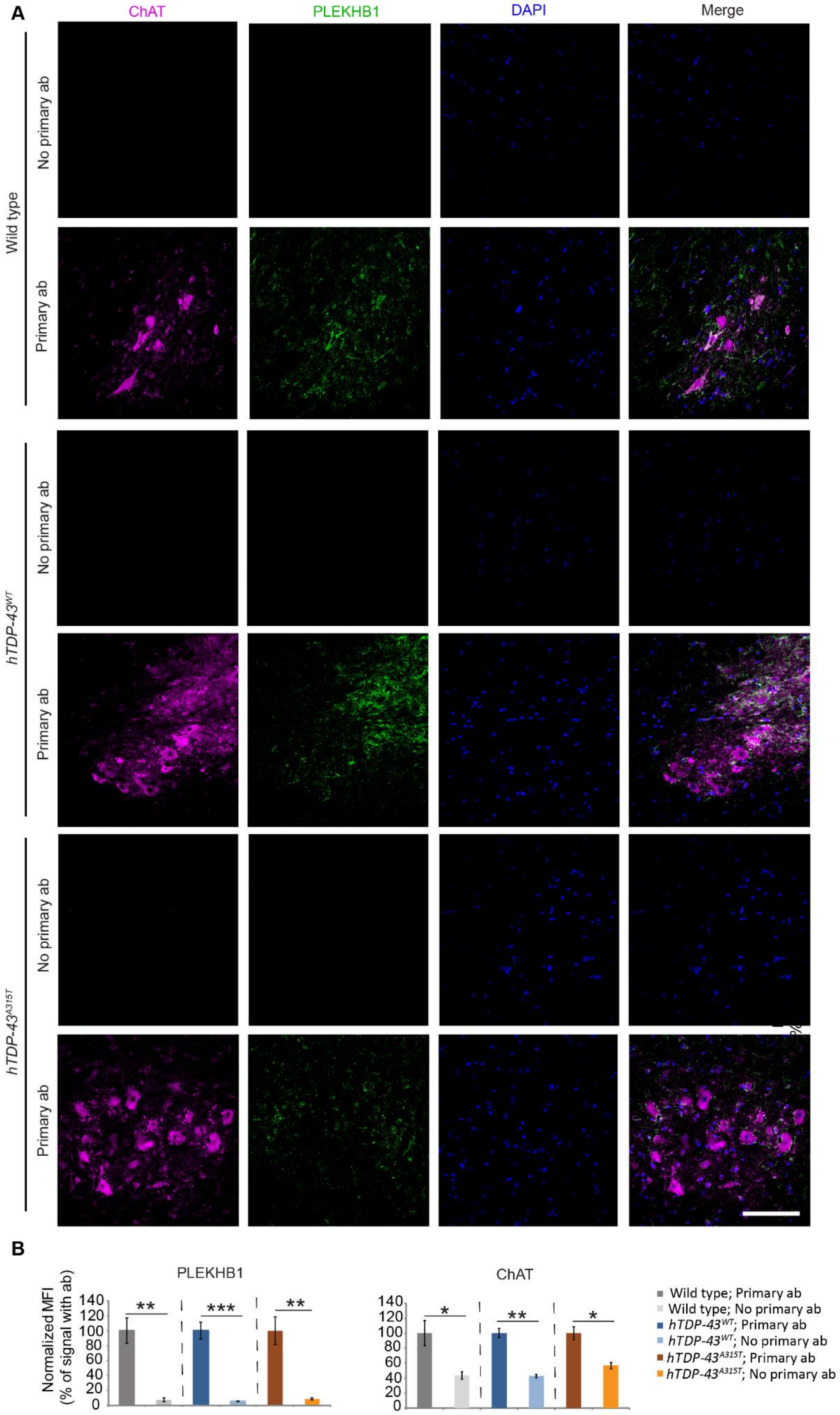
A
ChAT
PLEKHB1
DAPI
Merge
Wild type
No primary ab
Primary ab
hTDP-43^WT
No primary ab
Primary ab
hTDP-43^A315T
No primary ab
Primary ab
B
PLEKHB1
ChAT
Normalized MFI
(% of signal with ab)
0
20
40
60
80
100
120
140
**
***
**
*
**
*
Wild type; Primary ab
Wild type; No primary ab
hTDP-43^WT; Primary ab
hTDP-43^WT; No primary ab
hTDP-43^A315T; Primary ab
hTDP-43^A315T; No primary ab

Supp. Fig. 10. PLEKHB1 protein staining is primary antibody dependent. (A) Spinal cord staining of wild type, *hTDP-43*^{WT} and *hTDP-43*^{A315T}. Animals were tested for signal to noise (background) levels in the absence of primary antibodies. Images are representative of n=3 experiments. **(B)** Quantification of signal to noise for PLEKHB1 and ChAT antibodies. For each genotype, values for no primary control images (noise) are indicated as a percentage of signal in the presence of primary antibody (signal). All antibodies show significantly higher mean fluorescence intensity (MFI) when compared to the respective no primary antibody control. n=3 independent biological replicates; ab: antibody; two-tailed, two sample t-test; mean values +/- SEM are shown; *p<0.05, **p<0.01 and ***p<0.001; scale bar: 10µm.

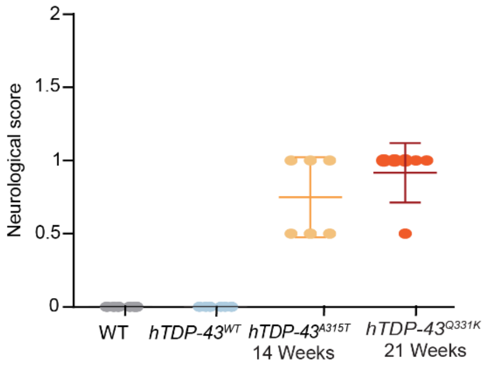

Supp. Fig. 11. Neurological phenotype of *hTDP-43*^{A315T} and *hTDP-43*^{Q331K} mice used for IHC analysis. Average neurological scores for mice used in IHC experiments. The *hTDP-43*^{A315T} (A315T) and *hTDP-43*^{Q331K} (Q331K) mice were 14 and 21 weeks of age, respectively. n=6 for A315T and for Q331K; mean values +/- SEM are shown.

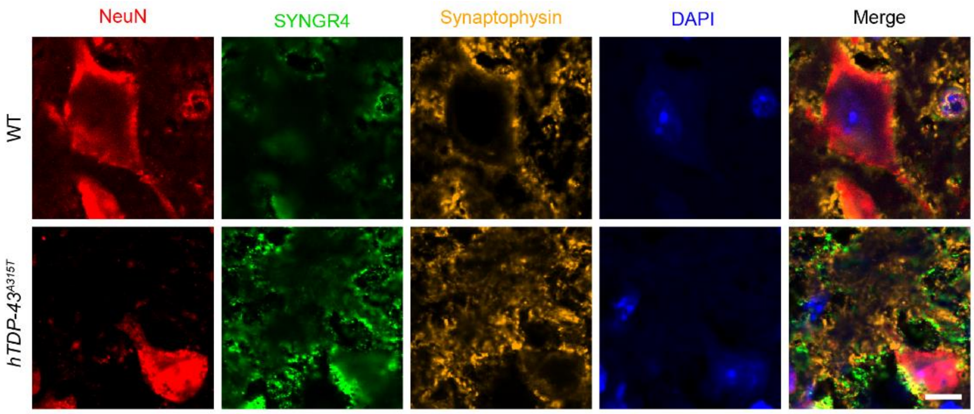

Supp. Fig. 12. SYNGR4 protein does not colocalize with synaptic vesicles in spinal cord. Spinal cord MNs in L3-5 were co-stained for SYNGR4 and the synaptic vesicle marker, Synaptophysin, as well as NeuN and DAPI. Representative confocal images from n=3 experiments are shown. Scale bars: 10µm.

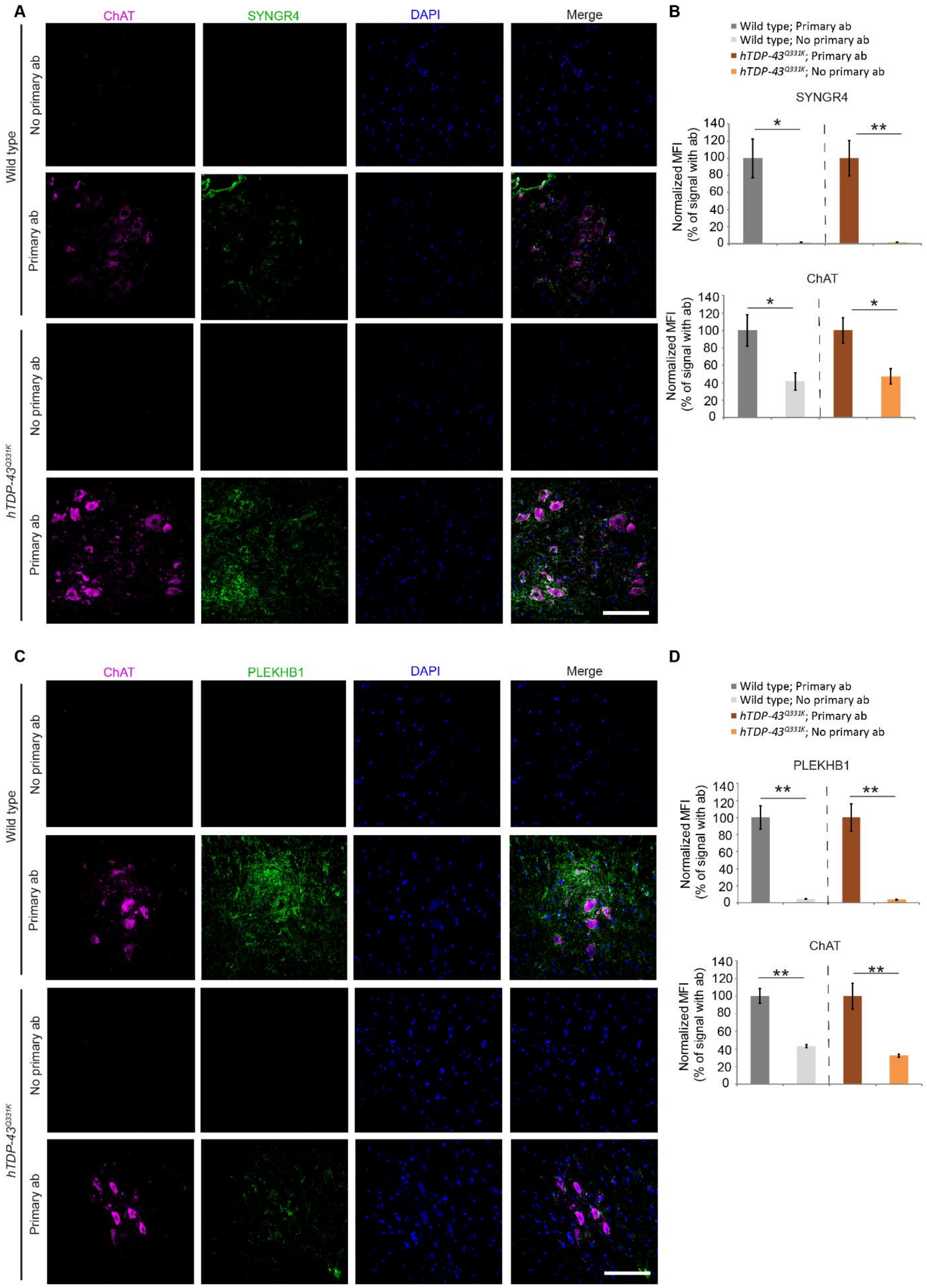

Supp. Fig. 13. PLEKHB1 and SYNGR4 protein staining is primary antibody dependent in the *hTDP-43*^{Q331K} line. (A) Spinal cord staining of wild type and *hTDP-43*^{Q331K}. Animals were tested for SYNGR4 and ChAT signal to noise (background) levels in the absence of primary antibodies. Images

are representative of n=3 experiments. **(B)** Quantification of signal to noise for SYNGR4 and ChAT antibodies. Values are indicated as percentage of signal relative to the presence of antibody per genotype. **(C)** Spinal cord staining of WT and *hTDP-43*^{Q331K}. Animals were tested for PLEKHB1 and ChAT signal to noise (background) levels in the absence of primary antibodies. Images are representative of n=3 experiments. **(D)** Quantification of signal to noise for PLEKHB1 and ChAT antibodies. Values are indicated as percentage of signal relative to the presence of antibody per genotype. All antibodies show significant mean fluorescence intensity (MFI) when comparing to the respective no primary antibody control. n=3; ab: antibody; two-tailed, two sample t-test; mean values +/- SEM are shown; *$p<0.05$ and **$p<0.01$; scale bar: 10µm.

SUPPLEMENTARY TABLES

Supp. Table 1. Mouse lines used in this study.

Short Name	Full strain name	Source	Reference
**C57BL/6J**	C57BL/6J	The Jackson Laboratory Stock No. 000664	*www.jax.org*
***Chat* bacTRAP**	B6J.Cg-Tg (Chat-EGFP/Rpl10a,Slc18a3) DW167Htz/J	The Jackson Laboratory Stock No. 030250	[37]
***hTDP-43*^{WT}**	B6J.Cg-Tg (Prnp-TARDBP)3Cptrc/J	The Jackson Laboratory Stock No. 016608	[44]
***hTDP-43*^{A315T}**	B6J.Cg-Tg (Prnp-TARDBP*A315T)95Balo/J	The Jackson Laboratory Stock No. 010700	[38]
***hTDP-43*^{Q331K}**	B6J.Cg-Tg (Prnp-TARDBP*Q331K)103Dwc/J	The Jackson Laboratory Stock No. 017933	[31]

Supp. Table 2. PCR primers used in this study.

Gene name	Forward primer (5´-3´)	Reverse primer (5´-3´)
Chat	CCA TTG TGA AGC GGT TTG GG	GCC AGG CGG TTG TTT AGA TAC A
Gapdh	TTG ATG GCA ACA ATC TCC AC	CGT CCC GTA GAC AAA ATG GT
Cnp1	TGC TTG ATG ATA CCA ACC ACG	GCT GGG CAC AGT CTA GTC G
Gfap	GTA AAG ACT GTG GAG ATG CGG GAT GGT TGA G	GTG CTG GTG TGG GTG GGA ACT GAG
L10a	CAT GAG CAG CAA AGT C	TAT TGG AGC ATC CTA ATA CA
Tex26	ACA AGA TGG CCC GGT ATA GGA	TGT CAC CTG ACT GGA GCT T
Plekhb1	AGA GTC AAG CCC CAC TTC TG	GAC TTT CCA GGA TGG AGT CG
Syngr4	CCT GGC AGA CAA TGA GAC TG	GAG GAC AAG ATG ACC AGG GA
Tia1	TGG ATA CAG CAG GAA ATG ACC	CAT TAT CTT CCG CCC ATT CA

Supp. Table 3. Differential gene expression at 14 weeks.

Supp. Table 4. Gene set enrichment analysis at 14 weeks.

Supp. Table 5. Differential gene expression at 9 weeks.

Supp. Table 6. Gene set enrichment analysis at 9 weeks.

Supp. Table 7. Overlap differential gene expression between 9 and 14 weeks.
